# The microbiome: a link between obesity and breast cancer risk

**DOI:** 10.3389/frmbi.2024.1394719

**Published:** 2024-05-03

**Authors:** Mohamed Gaber, Alana A. Arnone, Pierre-Alexandre Vidi, Katherine L. Cook

**Affiliations:** ^1^ Department of Cancer Biology, Wake Forest University School of Medicine, Winston-Salem, NC, United States; ^2^ Department of Surgery, Wake Forest University School of Medicine, Winston-Salem, NC, United States; ^3^ Integrative Physiology and Pharmacology, Wake Forest University School of Medicine, Winston-Salem, NC, United States; ^4^ Institut de Cancérologie de l’Ouest, Angers, France; ^5^ Atrium Health Wake Forest Baptist Comprehensive Cancer Center, Winston-Salem, NC, United States

**Keywords:** obesity, breast cancer, microbiome, inflammation, MAMP signaling

## Abstract

Globally, breast cancer is the leading cause of cancer incidence and mortality among all female cancers. Hereditary factors only account for 5-10% of breast cancers, highlighting the importance of non-hereditary factors, such as obesity. The increasing prevalence of obesity underscores the need to understand its contribution to breast cancer risk. Multiple mechanisms may mediate pro-carcinogenic effects of obesity, including altered adipokine levels, local and systemic inflammation, disruption of insulin and insulin-like growth factor signaling, increased estrogen levels, and alterations of the microbiome. In this review, we focus on the link between gut microbiome alterations and breast cancer risk in the context of obesity. First, we discuss how obesity influences the gut microbiome. Next, we describe the effect of such microbiome alterations on breast carcinogenesis, highlighting underlying molecular mechanisms. Finally, we review preclinical data on the interactions between host and bacteria, current challenges to study the obesity-microbiome connection, and future perspectives in this field.

## 1 Introduction

Since 1975, the global prevalence of obesity has tripled, with increases occurring across developed and developing countries. According to the latest World Health Organization (WHO) estimates, more than 1.9 billion adults were overweight and 650 million adults were classified as obese (W.H.O, 2021). Overweight and obesity reflect an excessive accumulation or abnormal distribution of fat and are classified according to the body mass index (BMI). BMI between 25 and 30 kg/m^2^ defines overweight whereas BMI ≥ 30kg/m^2^ corresponds to obesity. Over the past decades, trends toward increased intake of calorie-dense foods rich in fat and sugar, and decreased physical activity explain, in part, the obesity pandemic (W.H.O, 2021). Obesity is associated with chronic diseases such as type 2 diabetes, hypertension, dyslipidemia, cardiovascular diseases, non-alcoholic fatty liver disease, and 13 different cancer types, including postmenopausal breast cancer (Avgerinos et al., 2019; Włodarczyk and Nowicka, 2019; Dwivedi et al., 2020; Brown, 2021).

**Figure 1:**
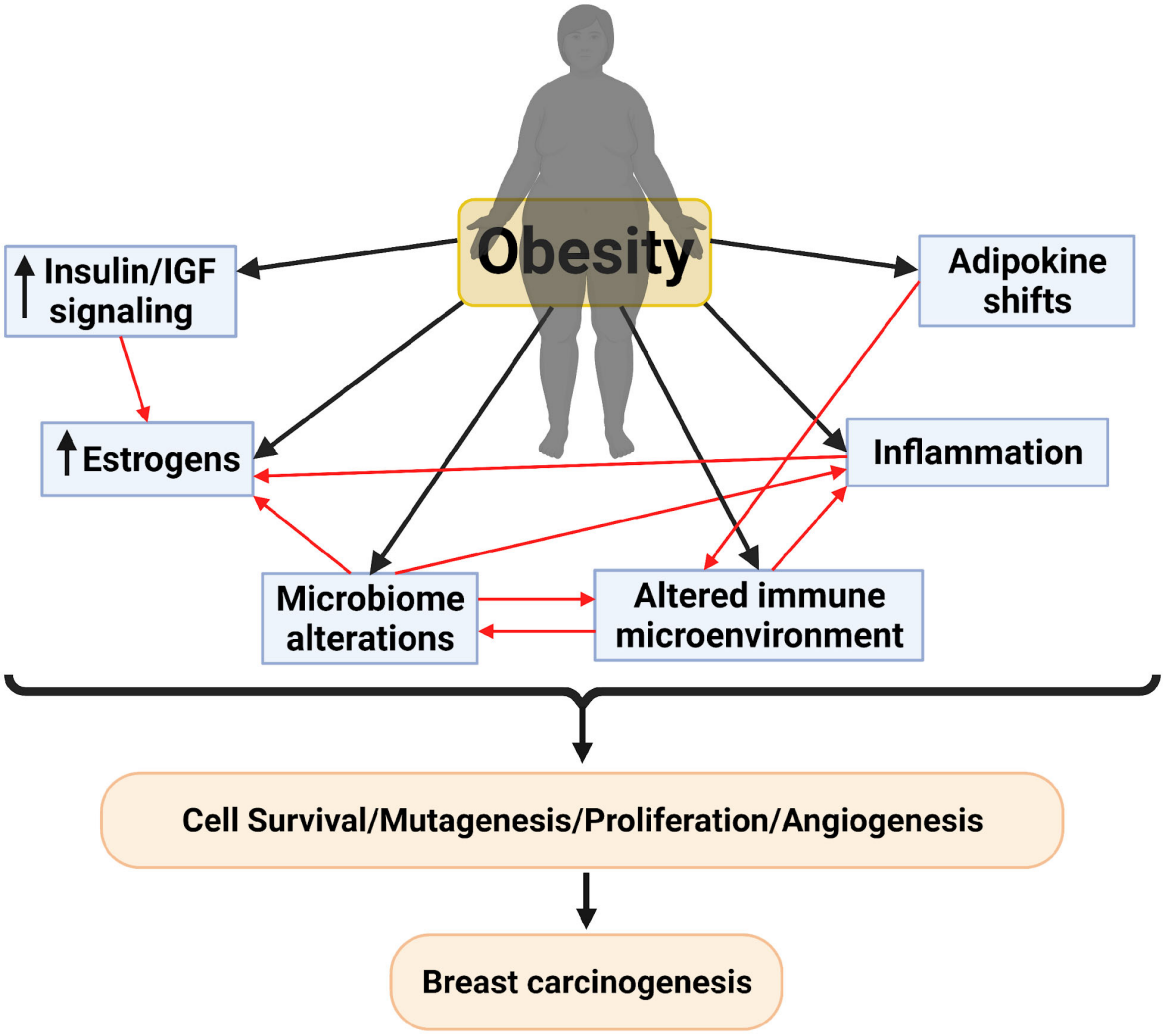
Proposed mechanisms for breast carcinogenesis mediated by obesity and the interplay between these different mechanisms. Created with BioRender.com.

Obesity increases the risk of developing postmenopausal breast cancer by up to 50% (Lauby-Secretan et al., 2016; Chan et al., 2019; Brown, 2021). Further, breast cancer patients (regardless of subtype and menopausal status) with obesity also have poorer overall survival, reduced response to chemotherapy and endocrine-targeted therapies, increased risk of local recurrence and metastasis, and often develop dose-limiting comorbidities (Ewertz et al., 2011; Rock et al., 2015; Iwase et al., 2016; Picon-Ruiz et al., 2017). Nevertheless, the mechanistic links between obesity, lipid metabolism, and breast cancer initiation and progression are still poorly characterized. The obesity-associated increase in both breast cancer risk and progression is thought to be regulated by several systemic and localized factors (Brown, 2021). These mechanisms include inflammation and immunosuppression, hypoxia of breast tissue, increased aromatase-mediated estrogen conversion, perturbations in the adipokines leptin and adiponectin, and changes in insulin signaling (Gunter et al., 2009; Ollberding et al., 2013; Himbert et al., 2017). Intriguingly, all these potential risk factors are also influenced by the gut microbiome (**Figure 1**), suggesting a critical relationship between microbes in the gut and obesity-driven breast cancer burden, which we will discuss in more detail in this review.

## 2 Alterations to the gut microbiome caused by diet-induced obesity

The gut microbiome is a dynamic and functional entity that is vital for the health of its host. It is shifted by host factors such as age, diet, lifestyle, xenobiotic agents, and disease state. Functionally, gut microbiota has an essential role in host physiology, including digestion of plant polysaccharides, biosynthesis of essential vitamins, detoxification of environmental pollutants, maintenance of the intestinal epithelial barrier function, enhancement of the immune function, and out-competition of pathological bacteria (Hansen and Sams, 2018; Heintz-Buschart and Wilmes, 2018; Rowland et al., 2018). The gut microbiome is composed of fungi, archaea, protists, viruses, and bacteria. The latter two account for >99% of the gut microbiome (Liang and Bushman, 2021; Zhang et al., 2022). Bacteroidetes and Firmicutes are the two major phyla of the gut microbiome, accounting for >90% of its composition (Plottel and Blaser, 2011; Tilg and Kaser, 2011). This distribution of bacteria is similar in mice, which are frequently used in gut microbiome studies. Mice and humans also share qualitative similarities in their gut microbiomes at the genus level (Krych et al., 2013). Non-human primates share even more similarities with the human microbiome; especially those in captivity and fed a human-like diet, suggesting that non-human primates may be a superior model to study the microbiome’s impact on health in humans (Nagpal et al., 2018).

Dysbiosis, or perturbations in the gut microbiome, is associated with the development of inflammatory, autoimmune, and malignant diseases, which can occur locally or at distant tissue sites. Many pathophysiologic conditions cause dysbiosis such as inflammatory bowel disease, diabetes mellitus, and obesity (Carding et al., 2015). Obesity alters the diversity and the relative abundance of microbes in the gut. Evidence supporting obesity-associated dysbiosis is from analyses of genetically obese leptin-deficient (*ob*/*ob*) mice. 16s ribosomal RNA (rRNA) sequencing revealed a major increase in the Firmicutes to Bacteroidetes (F/B) ratio (a marker of dysbiosis) of the ob/ob mice compared to their lean wild type (WT) siblings fed the same diet (Ley et al., 2005). Similar phyla-level changes were found in mice with diet-induced obesity (DIO) (Turnbaugh et al., 2008), as well as in obese humans (Ley et al., 2006). An unfavorable signature that has been frequently associated with obesity is the expansion in Proteobacteria phyla (Xu et al., 2022). It is imperative to mention that studies on mice with DIO showed a more consistent increase in the F/B ratio than studies on obese humans (Bisanz et al., 2019). This might be attributed to the experimental modeling in mice where the majority of studies have used chow as a control diet which has different dietary composition than the high fat diet (Dalby et al., 2017). Hence, this adds dietary composition differences on top of the adiposity differences between mice groups that could have confounded the microbiome sequencing results. Yet, in addition to the effect of diet, body adiposity within a dietary pattern further modified the gut microbiome in non-human primates, demonstrating that obesity alters the gut microbiome (Newman et al., 2021). Mice with DIO showed a marked decrease in microbial diversity in comparison to their lean counterparts (Turnbaugh et al., 2008). Human studies on obese individuals also revealed a marked decrease in microbial diversity (Le Chatelier et al., 2013).

While adiposity influences the gut microbiome, the inverse is also true. Mice with an intact gut microbiome showed a 42% increase in adiposity compared to germ-free mice, despite a 29% higher food intake by the germ-free mice. Accordingly, gut microbiota transplantation from conventionally-raised (lean) mice to germ-free mice caused a 60% increase in adiposity despite the decreased food intake (Bäckhed et al., 2004). Furthermore, germ-free mice were more resistant to DIO than conventionally raised mice, demonstrating how integral the microbiome is in the obesity pathogenesis process (Bäckhed et al., 2007). Interestingly, transplantation of gut microbiota from obese and lean mice caused differing degrees of adiposity in germ-free mice, with “obese microbiome” transplants causing a greater increase in adiposity than the “lean microbiome” transplants. The “obese microbiome” increased the capacity for energy harvest as demonstrated by gene enrichment for enzymes in pathways involved in galactose metabolism, starch/sucrose metabolism, and butanoate metabolism (Turnbaugh et al., 2006). Hence, obesity and gut microbiome perturbations have a two-way relationship and the perturbations to the gut microbiota caused by obesity are major drivers for obesity pathogenesis.

## 3 Gut microbiome alterations and breast carcinogenesis

Mounting evidence suggests a causative role of the gut microbiome in carcinogenesis. Although various microorganisms are associated with different cancer types, none to date were demonstrated to be causative for breast cancer (I.A.R.C., 2023). However, experiments with germ-free mice and rats revealed the tumor-promoting effects of the gut microbiome. Animals with an intact gut microbiome had more spontaneous, genetically-induced, and carcinogen-induced tumors compared to germ-free counterparts in various organs including the lung (Schreiber et al., 1972), liver (Dapito et al., 2012; Yoshimoto et al., 2013), skin (Sacksteder, 1976), colon (Grivennikov et al., 2012), and mammary gland (Mishra et al., 2021). Additionally, gut dysbiosis induced by obesity was associated with enhanced tumor growth and significant loss of gut microbial diversity in a murine triple-negative breast cancer (TNBC) model (Hossain et al., 2021). Moreover, multiple studies have shown that the gut microbiome is different in malignant breast disease, benign breast disease, and control. These observations are summarized in **Table 1**; which include all articles identified by performing a systematic search of PubMed articles published until February 22, 2024, for combinations of search terms: “Obesity”, “Gut microbiome”, and “Breast Cancer”. Mechanisms proposed for dysbiosis-associated breast cancer risk include alteration of normal tissue metabolism, induction of chronic inflammation, direct genotoxicity, and modulation of immune responses (Plottel and Blaser, 2011; Schwabe and Jobin, 2013; Argolo et al., 2018).

**Table 1:** Gut microbiome dysbiosis in breast cancer.

Study, Year, Country	Methodology	Comparison(s)	Differentially regulated microbes in breast cancer
Zhu, Jia et al., 2018, China (Zhu et al., 2018)	Metagenomic sequencing	44 postmenopausal BrCa patients *vs*. 46 postmenopausal HCs	**↑** *Escherichia coli* and *Prevotella amnii …* etc. **↓** *Eubacterium eligens* and *Lactobacillus vaginalis …* etc.
		18 premenopausal BrCa patients *vs*. 25 premenopausal HCs	**↔**
Bertazzoni, E. Minelli et al., 1990, Italy (Minelli et al., 1990)	Simple culturing, morphological and biochemical analysis	18 BrCa patients *vs*. 30 HCs	**↑** *Bacteroides*, *Clostridia*, and anaerobic *Lactobacilli …* etc.
Bobin-Dubigeon, Christine et al., 2021, France (Bobin-Dubigeon et al., 2021)	V3 and V4 16S rRNA sequencing	25 BrCa patients *vs*. 30 HCs	**↑** *Firmicutes* phylum, *Blautia* genus, and *Clostridium clusters IV* and *XIVa* **↓** *Bacteroidetes* phylum
Shrode, Rachel L. et al., 2023, USA (Shrode et al., 2023)	V3 and V4 16S rRNA sequencing	24 BrCa patients *vs*. 23 HCs	**↑** F/B ratio, *Oscillospiraceae* family, *Actinomyces* genus, *Blautia* genus, and *Eggerthella lenta …* etc. **↓** *Alistipes* genus, *Faecalibacterium prausnitzii*, Lachnoclostridium edouardi, and Lachnospira pectinoshiza … etc.
Aarnoutse, Romy et al., 2021, Netherlands (Aarnoutse et al., 2021)	V4 16S rRNA sequencing	81 BrCa patients vs. 67 HCs	**↔**
Jiang, Yonglan et al., 2023, China (Jiang et al., 2023)	Full length16S rRNA sequencing	43 BrCa patients vs. 30 HCs	**↑** *Firmicutes* phylum, Lachnospira genus, and *Coprococcus* genus … etc. **↓** *Bacteroidetes* phylum, *Bacteroides* genus, *Veillonella* genus, and *Eggerthella* genus … etc.
Byrd, Doratha A. et al., 2021, Ghana (Byrd et al., 2021)	V4 16S rRNA sequencing	379 BrCa patients *vs*. 414 HCs	**↑** *Bacteroides* **↓** *Romboutsia*, *Pseudobutyrivibrio*, and *Coprococcus 2*…etc.
		102 non-malignant breast disease patients *vs*. 414 HCs	**↑** *Bacteroides*
		379 BrCa patients *vs*. 102 non-malignant breast disease patients	**↔**
He, Chuan et al., 2021, China (He et al., 2021)	V3 and V4 16S rRNA sequencing	54 premenopausal BrCa patients *vs*. 28 premenopausal HCs	**↑** F/B ratio, *Synergistetes* phylum, *Clostridium*_IV, *Eubacterium*, and *Terrisporobacter …* etc. **↓** *Acidobacteria*, *Nitrospirae*, *Fusobacteria* and *Cyanobacteria* phyla, *Allisonella*, *Megasphaera*, *Pediococcus*, *Fusobacterium*, and *Enhydrobacter …* etc.
Ma, Zhihjun et al., 2022, China (Ma et al., 2022)	V3 and V4 16S rRNA sequencing	26 BrCa patients *vs*. 20 HCs	**↑** *Escherichia*, *Peptoniphilus*, *Bilophila*, *Lactobacillus*, and *Porphyromonas* **↓** *Faecalibacterium*, *Lachnospiracea_incertae_sedis*, *Collinsella*, *Alistipes*, and *Anaerofilum …* etc.
		20 non-malignant breast disease patients *vs*. 20 HCs	**↑** *Escherichia*, *Peptoniphilus*, *Coprobacillus*, *Lactobacillus*, and *Porphyromonas* **↓** *Collinsella*, *Alistipes*, *Megamonas*, and *Butyricimonas …* etc.
Hou, Ming-Feng et al., 2021, Taiwan (Hou et al., 2021)	V3 and V4 16S rRNA sequencing	100 premenopausal BrCa patients *vs*. 50 premenopausal HCs	**↑** *Sutterella*, *Haemophilus*, and *Bacteroides …* etc. **↓** *Actinobacteria* phylum, *Streptococcus*, *Bifidobacterium*, and *Akkermansia …* etc.
		100 postmenopausal BrCa patients *vs*. 17 postmenopausal HCs	**↑** *Proteobacteria* phylum, *Sutterella*, and *Haemophilus …* etc. **↓** *Verrucomicrobia* phylum, *Akkermansia*, and *Streptococcus …* etc.
Goedert, James J. et al., 2015, USA (Goedert et al., 2015)	V3 and V4 16S rRNA sequencing	48 postmenopausal BrCa patients *vs*. 48 postmenopausal HCs	**↑** *Clostridiaceae*, *Faecalibacterium*, and *Ruminococcaceae …* etc. **↓** *Dorea* and *Lachnospiraceae …* etc.
Goedert, James J. et al., 2018, USA (Goedert et al., 2018)	V3 and V4 16S rRNA sequencing	48 postmenopausal BrCa patients *vs*. 48 postmenopausal HCs	**↑** IgA-positive *Parasutterella* **↓** IgA-positive *Oscillibacter*, IgA-negative *Alistipes indistinctus*, and IgA-negative *Ruminococcus …* etc.
Ma, Ji et al., 2020, China (Ma et al., 2020)	16S rRNA sequencing (hypervariable region unmentioned)	25 BrCa patients *vs*. 25 non-malignant breast disease patients	**↑** *Proteobacteria*, *Verrucomicrobia* and *Actinobacteria* phyla … etc. **↓** *Firmicutes* and *Bateroidetes* phyla, *Subdoligranulum*, and *Faecalibacterium prausnitzii …* etc.
Smith, K.S. et al., 2021, USA (Smith et al., 2021)	V4 16S rRNA sequencing	14 overweight/obese BrCa patients *vs*. 14 matched overweight/obese HCs	**↑** *Allobaculum* **↓** *Phenylobacterium*, *Rhodospirillum*, *Balneimonas*, *Rubellimicrobium*, *Aquabacterium*, *Vogesella*, and *Lysobacter*
Yang, Peidong et al., 2021, China (Yang et al., 2021)	V4 16S rRNA sequencing	83 BrCa patients *vs*. 19 non-malignant breast disease patients	**↑** *Citrobacter* **↓** *Clostridium*, *Lachnospira*, and *Faecalibacterium …* etc.

BrCa, Breast Cancer; HCs, Healthy Controls; **↑**, Increase; **↓**, Decrease; **↔**, No Difference.

Changes in gut microbiome composition and diversity have been documented in breast cancer. A pilot study by Bertazzoni et al. performed using simple culturing techniques (predating current sequencing methods) found significant increases in *Bacteroides*, *Clostridia*, and anaerobic *Lactobacilli* in breast cancer patients compared to healthy controls (Bertazzoni et al., 2006). Later studies of the gut microbiome using 16S rRNA or metagenomic sequencing affirmed the initial findings of dysbiosis in breast cancer patients (**Table 1**). Most studies show a significant reduction in alpha diversity which represents the compositional complexity of a single sample (intra-sample heterogeneity, **Box 1**), in breast cancer patients compared to cancer-free controls (**Table 2**). This is consistent with loss of microbiome richness and evenness, which has been associated with unhealthy gut environments, aging, and disease state (Hou et al., 2021). For beta diversity, which corresponds to the taxonomical or phylogenetic differences between samples (inter-sample heterogeneity, **Box 1**), an opposite trend is observed where most of the studies showed significant increases in beta diversity (**Table 3**). This reflects an increased heterogeneity between samples of breast cancer patients compared to healthy women. This may be due to inter-individual differences in breast cancer stages, grades, hormone receptor status, HER2 status, and proliferation levels which are factors that differentially impact the gut microbiome (Luu et al., 2017; Wu et al., 2020; Yang et al., 2021). For instance, clinical stages II and III had significantly higher levels of *Bacteroidetes*, *Blautia*, *Clostridium coccoides*, and *Faecalibacterium prausnitzii* compared to stages 0 and I (Luu et al., 2017). Also, patients with non-malignant breast disease had an altered microbiome, different from that of breast cancer patients (Luu et al., 2017). Another potential confounder that is often overlooked in these comparisons is dietary differences that might have existed between breast cancer and cancer-free subjects (Ma et al., 2020).

Box 1 **Key terms and concepts in microbiome research.**

**Table:** 

Concept	Definition
Operational taxonomic units (OTUs)	Clusters of microorganisms with shared DNA sequence similarity based on a specific taxonomic marker gene like the nine hypervariable regions of the16S rRNA genes (V1-V9) widely used to classify bacterial taxa.
Alpha (α) diversity	“A metric that reflects the structure of a microbial community. It takes into account the richness (number of taxa) and/or evenness (the relative abundances of those taxa) within a microbial sample. Commonly used metrics are Observed Richness, PD, Chao1, Shannon, and Simpson.”*
Beta (β) diversity	“A metric that reflects the differences in the composition between microbial samples. Commonly used metrics are weighted UniFrac, unweighted UniFrac, and Bray Curtis”.
Observed Richness	“It estimates the number of observed taxa/OTUs”.
PD	“It is a phylogenetically weighted measure of richness. It is the sum of the lengths of all those branches on the phylogenetic tree that span the members of the set”.
Chao1	“It is an abundance-based nonparametric estimator of taxa richness. This index gives more weight to the low-abundance taxa”.
Shannon	“Shannon’s index *H* is an estimator of taxa diversity, combining richness and evenness. This index places a greater weight on taxa richness”.
Simpson	“Simpson’s index *D* is an estimator of taxa diversity, combining richness and evenness. This index considers taxa evenness more than taxa richness”.
Bray Curtis	“It measures the compositional dissimilarity between the microbial communities of two samples. This index ranges between 0 (the two samples share all taxa) and 1 (the two samples do not share any taxa).”
Unweighted and weighted UniFrac	“UniFrac distances between two samples take into account the phylogenetic tree and thus phylogenetic distances between community members. In unweighted, the distance is calculated as the fraction of the branch length, and in weighted UniFrac, branch lengths are weighted by the relative abundance of sequences”.

*Definitions are summarized from this ref (Kers and Saccenti, 2021). The reader is referred to it for more details.

**Table 2:** Alpha diversity of the gut microbiome in breast cancer.

Study	Indices used^*^	Directionality in BrCa patients (vs. cancer-free subjects)
Bobin-Dubigeon, Christine et al. (Bobin-Dubigeon et al., 2021)	Chao1 and Shannon	**↓**
Ma, Zhihjun et al. (Ma et al., 2022)	Sobs and Chao1	**↓**
Byrd, Doratha A. et al. (Byrd et al., 2021)	Shannon, Observed richness, and PD	**↓**
Aarnoutse, Romy et al. (Aarnoutse et al., 2021)	Shannon and Observed richness	**↔**
Shrode, Rachel L. et al. (Shrode et al., 2023)	Chao1	**↔**
Hou, Ming-Feng et al. (Hou et al., 2021)	Shannon	**↓** (Pre-menopause) **↔** (Post-menopause)
Goedert, James J. et al. (Goedert et al., 2015)	Chao1, PD, Shannon, and Observed richness	**↓**
Goedert, James J. et al. (Goedert et al., 2018)	Chao1, PD, Shannon, and Observed richness	**↓**
Ma, Ji et al. (Ma et al., 2020)	PD and Observed richness	**↓**
He, Chuan et al. (He et al., 2021)	Shannon, Simpson, Observed richness, and Pielou’s evenness	**↔**
Jiang, Yonglan et al. (Jiang et al., 2023)	Chao1, Shannon, and Ace	**↑**

BrCa, Breast Cancer; HCs, Healthy Controls; PD, Phylogenetic Diversity; **↑**, Increase; **↓**, Decrease; **↔**, No Difference. *The reader is referred to Box 1 for the definition of each index.

**Table 3:** Beta diversity of the gut microbiome in breast cancer.

Study	Indices used^*^	Directionality in BrCa patients (vs. cancer-free subjects)
Ma, Zhihjun et al. (Ma et al., 2022)	Unweighted and weighted UniFrac	**↑**
Byrd, Doratha A. et al. (Byrd et al., 2021)	Unweighted UniFrac	**↑**
Goedert, James J. et al. (Goedert et al., 2015)	Unweighted UniFrac	**↑**
Ma, Ji et al. (Ma et al., 2020)	Unweighted UniFrac	**↑**
Zhu, Jia et al. (Zhu et al., 2018)	Jensen-Shannon divergence	**↑** (Post-menopause) **↔** (Pre-menoapuse)
Goedert, James J. et al. (Goedert et al., 2018)	Unweighted and weighted UniFrac, and Bray Curtis	**↔**

BrCa, Breast Cancer; HCs, Healthy Controls; **↑**, Increase; **↓**, Decrease; **↔**, No Difference. *The reader is referred to Box 1 for the definition of each index.

During menopause, the depletion of cycling estrogens can adversely impact physiological systems, including gut health (Peters et al., 2022). A comparison of the microbiome of non-obese pre vs. postmenopausal women (without breast cancer) found significant differences in β-diversity, demonstrating that menopause shifts the gut microbiome. Furthermore, postmenopausal women also have higher Firmicutes proportional abundance, higher F/B ratio, and increased levels of pro-inflammatory cytokines, including interleukin-6 (IL-6) and monocyte chemoattractant protein-1 (MCP-1). These differences could not be explained by the age difference since they were not observed in male control groups of the same age. Moreover, subjects were matched for their BMI and nutritional background. Hence, this strongly suggests the fundamental interactions between estrogen, microbiota, and inflammation (Santos-Marcos et al., 2018).

Several studies show altered gut microbiota populations in breast cancer patients depend on menopausal status. Bertazzoni et al. (2006) divided the breast cancer study participants according to their menopausal status and found that the genera and species cultured from each group were remarkably different from one another and from the healthy controls. Another study found significant differences in gut microbiota composition and diversity in postmenopausal (vs. matched healthy controls) but not in premenopausal women (Zhu et al., 2018). In particular, this group found a positive correlation between *Shewanella putrefaciens* and *Erwinia amylovora* with estradiol (p< 0.05) in postmenopausal patients. This is consistent with gut microbiota interactions with estrogen metabolism, suggesting a potential biomarker for breast cancer. *Roseburia inulinivorans*, a butyrate-producing bacteria, was found to be lower in postmenopausal breast cancer patients. Butyrate acts as an anti-inflammatory agent, by inhibiting the activation of nuclear factor-κB (NF-κB) in intestinal epithelial cells (Inan et al., 2000). Therefore, this reduction in *R. inulinivorans* may indicate postmenopausal women are more prone to inflammation and therefore at higher risk of breast carcinogenesis (Zhu et al., 2018). Another study demonstrated a significant difference in β-diversity between breast cancer patients and age-matched controls as well as a significant reduction in α-diversity in the premenopausal breast cancer group compared to controls (Hou et al., 2021). However, while premenopausal and postmenopausal patients had similar BMIs, BMIs were not provided for the age-matched controls. These comparisons may therefore not be controlled for adiposity. Using functional pathways analysis, the gut microbiota of premenopausal breast cancer patients showed enrichment in steroid-related aromatic and androstenedione degradation, which may result in DNA damage induction and, subsequently, breast cancer development (Heikkinen et al., 2015; Hou et al., 2021). Moreover, gut microbes of postmenopausal breast cancer patients showed enrichment in chemical carcinogenesis and aldosterone-related pathways. This could be attributed to the lower estrogen levels post-menopause which has been shown to increase aldosterone levels and, thus, may increase breast cancer risk (Rigiracciolo et al., 2016; Hou et al., 2021). Different gut microbiome compositions in premenopausal women with vs. without breast cancer were confirmed by another study (He et al., 2021). Collectively, these studies show that the gut microbiome is differentially regulated in breast cancer based on menopausal status. Yet, discrepancies in the altered microbes and diversity changes call for further investigations. Differences in other variables such as obesity status, race/ethnicity, age, diet, environmental exposures, sequencing methodology, and sample size are a few of the potential confounders.

## 4 Pro-carcinogenic effects of the gut microbiome in obesity

Few clinical studies have investigated the role of obesity-modulated gut microbiome in breast cancer. Luu et al. found that the gut microbiome composition in breast cancer patients differs according to BMI (Luu et al., 2017). The bacterial load was lower in obese/overweight patients compared to patients with normal weight. Among other differences, the abundance of *Faecalibacterium prausnitzii*, was significantly lower in the obese/overweight group. Interestingly, these bacteria produce butyrate, an anti-inflammatory short-chain fatty acid (SCFA), which will be discussed in detail below. In another study, breast cancer patients in the obese/overweight category showed significant enrichment of *Clostridiaceae* family and *Akkermansia* genus, and a significant reduction of *Lactobacillus* and *Streptococcus* genera (Wu et al., 2020). Total body fat also impacted the microbiome; patients with higher body fat had fewer detectable operational taxonomic units (OTUs, **Box 1**) and lower alpha diversity. These patients also had significant enrichment in *Clostridium* genus, and *Lachnospira* genus, and a significant reduction in *Catenbacterium* genus. These findings raise the question of whether differences in the gut microbiome between BMI or body fat categories can explain the worse prognosis and lower survival of obese breast cancer patients. A consensus on how obesity may influence microbial populations in the context of breast cancer has not been reached, and further investigations are needed.

**Figure 2:**
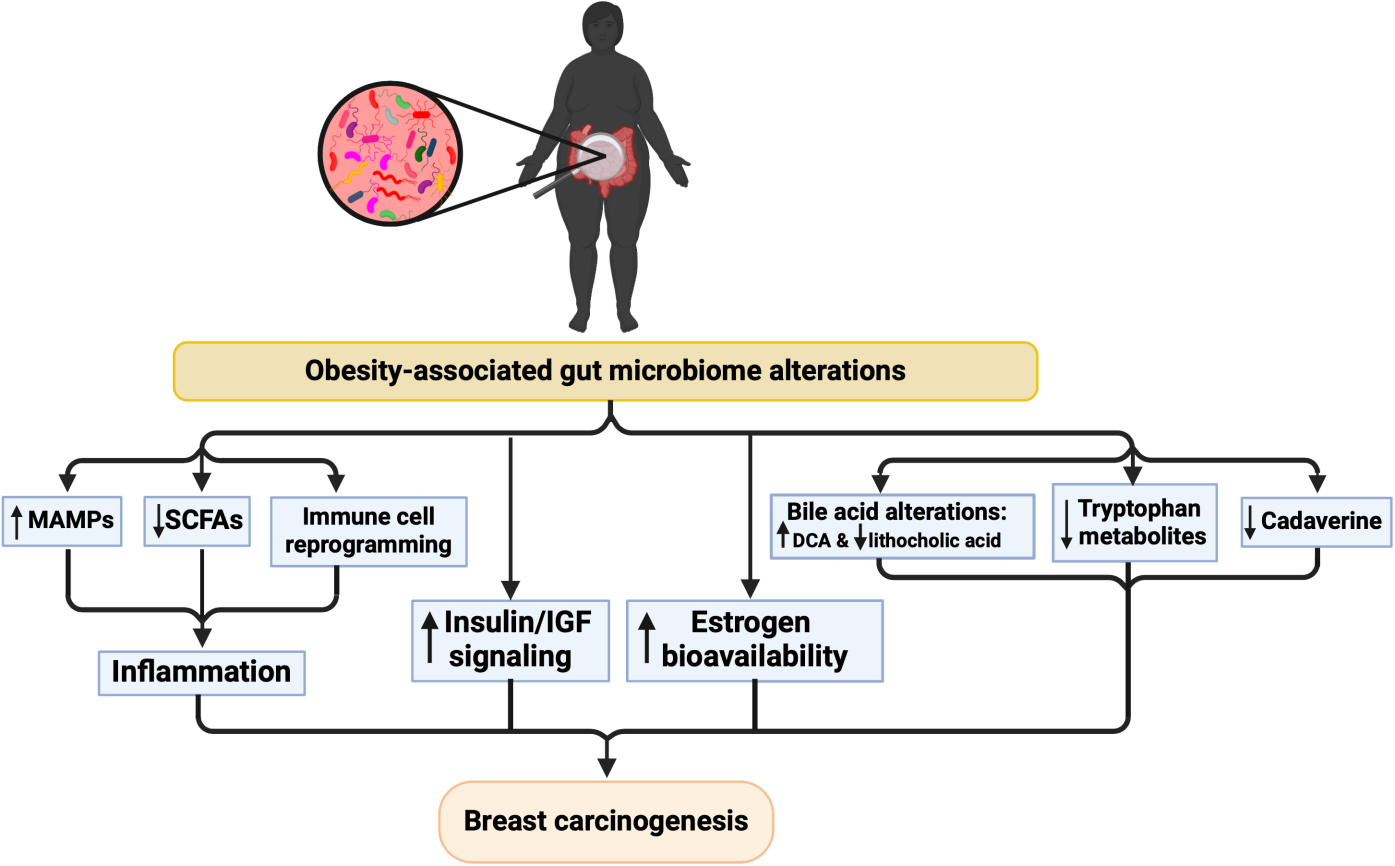
Proposed mechanisms underlying the relationship between microbiome alterations and breast cancer risk in the context of obesity. Created with BioRender.com.

The gut microbiomes of obese/overweight breast cancer patients were compared to the microbiomes of BMI-matched cancer-free women by Smith et al. (2021). While there was no significant difference in alpha or beta diversity, differences in gut microbiota were observed. Compared to BMI-matched cancer-free women, bacterial genera such as *Phenylobacterium* and *Balneimonas* were significantly reduced in obese/overweight breast cancer patients, while *Allobaculum* was significantly enriched. Taken together, these findings indicate a two-way relationship between tumorigenesis and gut microbiome composition, where the obesity-modulated microbiome increases breast tumorigenesis and the presence of a tumor imposes a selective pressure on the gut microbiome in obese patients. The latter is corroborated by an animal study on tumor-bearing and tumor-free obese mice, where the presence of the tumor modified the gut microbiome (Hossain et al., 2021). This is true in lean mice as well, where the presence of mammary tumors perturbs microbiome composition, compromises intestinal barrier function, increases translocation of gut bacteria, and induces systemic inflammation (Loman et al., 2022). Additionally, high-risk obese individuals may possess a pro-tumorigenic microbial signature prior to breast cancer development. Using a chemical mammary carcinogenesis model and manipulation of the gut microbiome with fecal microbiota transplants (FMT), we showed that intestinal microbes derived from lard-fed (obese) mice decreased tumor-free survival in lean animals (Soto-Pantoja et al., 2021). Reciprocally, obese mice benefited from FMT from lean animals, with lower tumor burden and increased survival. In a murine TNBC model, FMT from non-tumor bearing mice with DIO increased tumor growth in recipient mice (Bawaneh et al., 2022). These results demonstrate that the pro-tumorigenic effects of the lard diet are, at least in part, caused by shifts in the gut microbiome. Further research is required to determine whether obesity-mediated dysbiosis is a cause and/or a consequence of breast tumorigenesis. Potential mechanisms linking obesity, dysbiosis, and breast carcinogenesis are illustrated in **Figure 2** and discussed in the following sections.

### 4.1 Inflammation

Altered gut microbial composition and lower microbial diversity in obese subjects are associated with higher inflammation, implicating gut microbiota in low-grade inflammation, contributing to breast cancer development (Scheithauer et al., 2020). Consistently, differences in the gut microbiome between breast cancer patients and healthy controls (or between breast cancer patients and subjects with non-malignant breast disease) also correlate with differences in inflammation markers, such as increased expression of inflammatory cytokines (Tzeng et al., 2021) and enrichment of virulence factors such as the iron complex transport system and increased lipopolysaccharide (LPS) biosynthesis (Toumazi et al., 2021). The iron complex transport system increases pathogen abundance and induces intestinal inflammation (Jaeggi et al., 2015). Therefore, obesity-associated gut dysbiosis can lead to increased levels of pro-inflammatory cytokines promoting inflammation, and thereby contributing to breast cancer development. While further research is needed to understand the mechanisms involved, several mechanisms may contribute to inflammation via the microbiome in obesity, such as increased production or bioavailability of microbe-associated molecular patterns (MAMPs), decreased SCFA production or bioavailability, and reprogrammed immune microenvironment, which are discussed below.

#### 4.1.1 Microbial-associated molecular patterns

**Figure 3:**
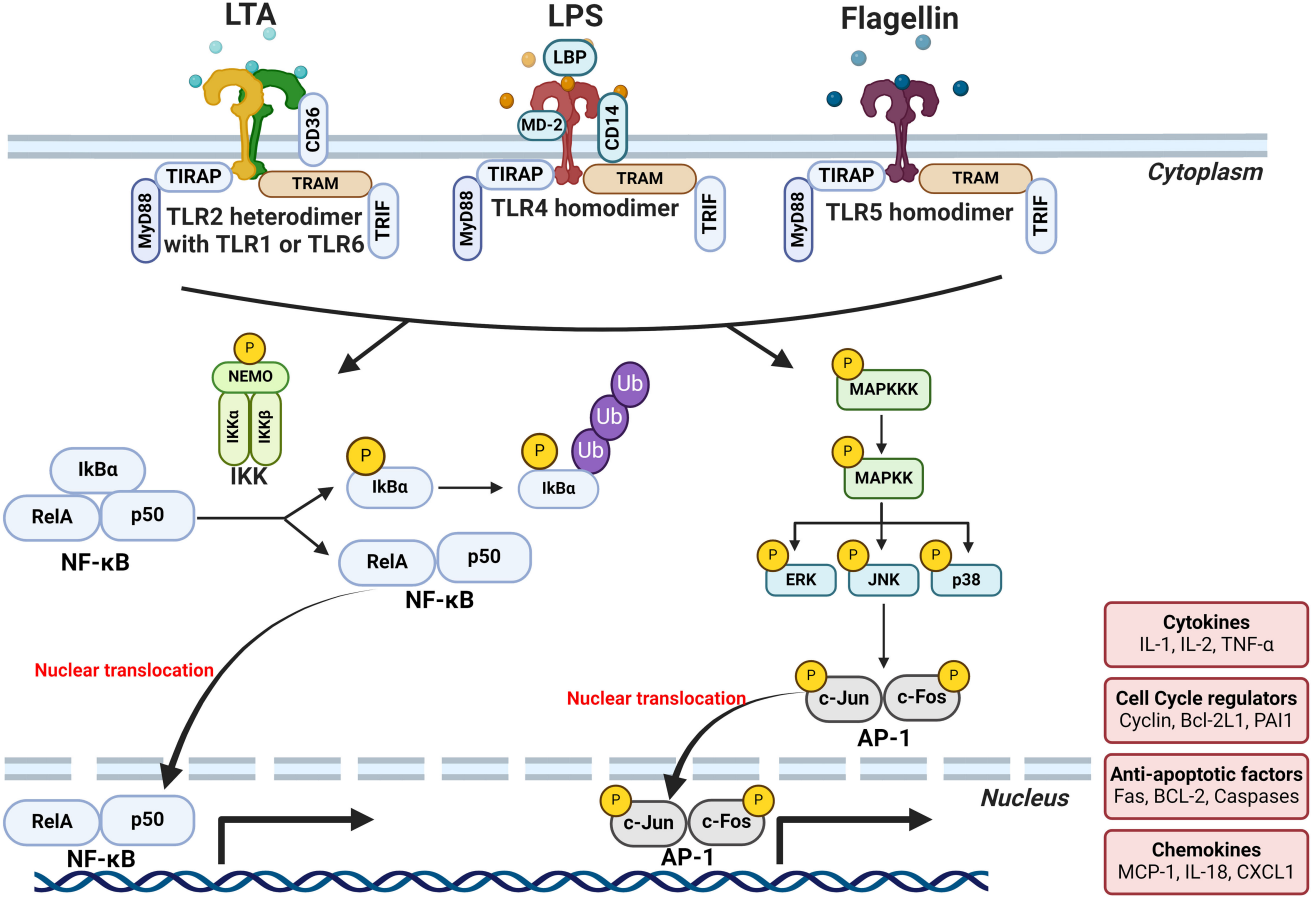
Mechanisms by which MAMPs may cause breast tumor-promoting inflammation. Binding of MAMPs (LTA, LPS and flagellin) to PRRs leads to the activation of transcription factors such as NFκB and AP-1 that induce the expression of several pro-inflammatory effectors. LPS and flagellin binding causes the homodimerization of their respective receptors, TLR4 and TLR5, respectively. While LTA binding causes the heterodimerization of TLR2 with either TLR1 or TLR6. The affinity of receptor-ligand interactions depends on a repertoire of ligand-binding proteins or accessory molecules that aid the dimerization of TLR4 and the subsequent signal transduction. For instance, LPS-binding protein (LBP), CD14, and myeloid differentiation protein 2 (MD-2) all interact with and enhance the LPS-TLR4 binding. While CD36 aids LTA-TLR2 binding. MAMP-TLR binding activates the TLR intracellular domains which then act through the binding of myeloid differentiation primary response gene 88 (MyD88) or TIR-domain-containing adapter-inducing interferon-β (TRIF) adaptor molecules to initiate signaling. Signal transduction subsequently leads to the translocation of NF-κB dimers such as RelA/p65 to the nucleus and their binding to the κB consensus motifs found in many gene promoters. Activation of TLRs also leads to the activation of the different members of mitogen-activated protein kinase (MAPK) family such as p38 and Jun N-terminal kinase (JNK). Activation of these MAPK members, in turn, activate the transcription of AP-1 monomers and enhances their transcriptional activity. The end result of this transcriptional activation of NFκB and AP-1 is the expression of a wide array of inflammatory cytokines and chemokines such as TNF-α, IL-1b, IL-6, IL-8, and IFN-γ creating an inflammatory milieu. Created with BioRender.com.

MAMPs are small molecular motifs conserved within a class of microorganisms that are recognized by pattern-recognition receptors (PRRs) and play a key role in innate immunity. LPS, the prototypical MAMP, is an essential structural component on the outer membranes of Gram-negative bacteria. Other MAMPs include lipoteichoic acid (LTA), an essential structural component in the cell wall of Gram-positive bacteria, and flagellin which is a structural component of the locomotory organ of flagellated bacteria. MAMPs are recognized by a wide array of PRRs. For example, LPS, LTA, and flagellin are recognized by membrane-bound toll-like receptors (TLR) 4, 2, and 5, respectively. Other PRRs include nucleotide-binding oligomerization domain-like receptors (NLRs) and retinoic acid-inducible gene-I-like receptors (RLRs) which are cytoplasmic PRRs (Murphy et al., 2017). Binding of MAMPs to PRRs leads to the activation of transcription factors such as NFκB and activator protein 1 (AP-1) that induce the expression of several pro-inflammatory effectors, including tumor necrosis factor (TNF)-α, interleukin (IL)-1b, IL-6, IL-8, and interferon (IFN)-γ (Boulangé et al., 2016) (**Figure 3**).

##### 4.1.1.1 LPS

**Figure 4:**
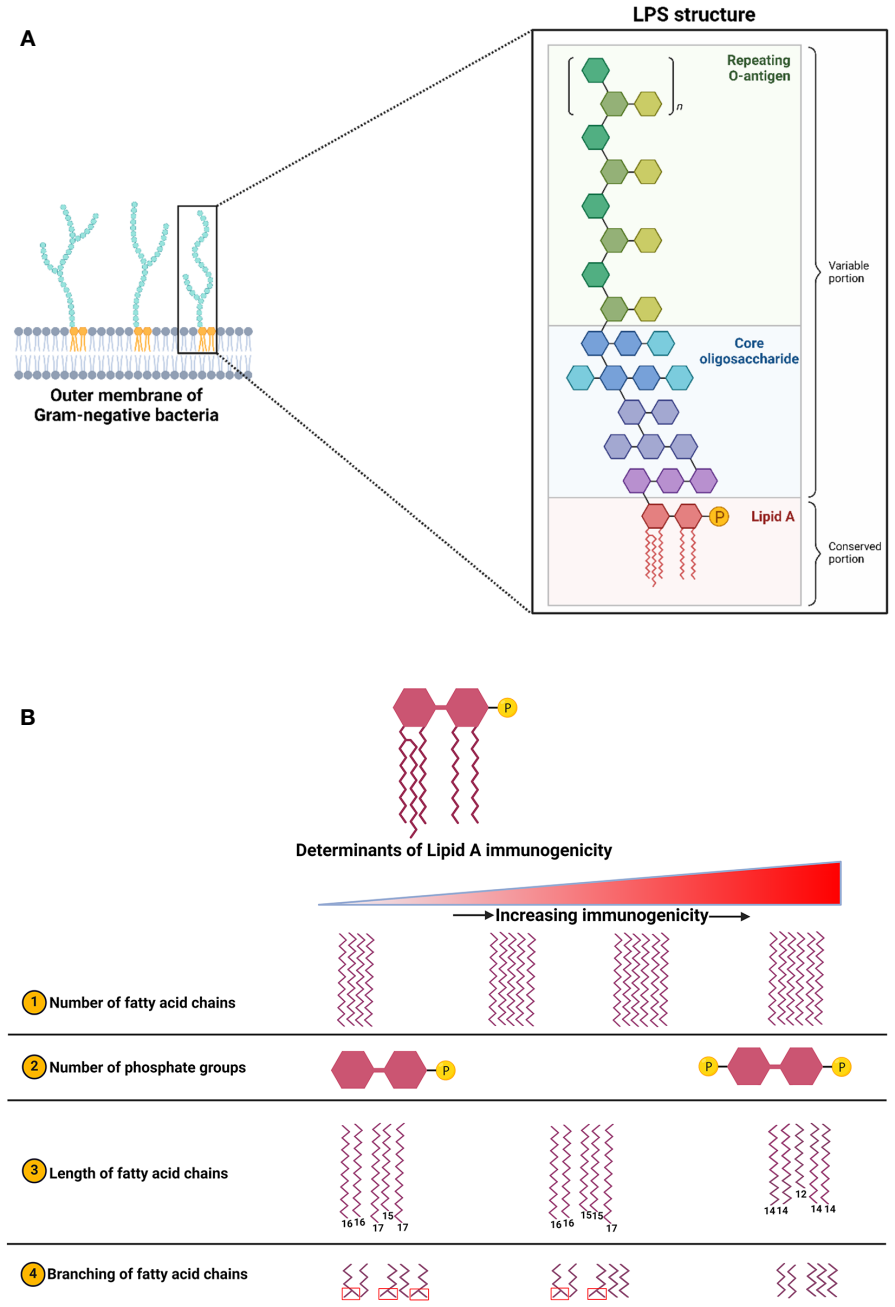
**(A)** Structure of LPS which is composed of three main blocks: Lipid A (innermost), core oligosaccharide, and O-antigen (outermost). **(B)** Factors influencing LPS immunogenicity which is determined by the structural features of its lipid A moiety. This figure is adapted and expanded from this ref Berezow et al. (2009). Created with BioRender.com.

One of the most-studied and classical examples of MAMP signaling is the activation of TLR4 by LPS. LPS binding causes homodimerization of TLR4 which then acts either through myeloid differentiation primary response gene 88 (MyD88) or TIR-domain-containing adapter-inducing interferon-β (TRIF) adaptor molecules to initiate signaling (**Figure 3**). Gene expression analysis of tumor tissues suggests the involvement of TLR4 signaling in breast tumorigenesis. Downregulation of TLR4 expression was observed in breast tumors, whereas MYD88, NF-κB, and other downstream genes were upregulated compared to healthy tissues (Xuan et al., 2014; Tzeng et al., 2021). Downregulation of the TLR4 receptor in breast tumors could be an adaptive response to prolonged exposure to LPS. Indeed, functional features of genes expressed by gut microbiota in breast cancer subjects showed enrichment in “LPS biosynthesis” pathways when compared to healthy controls (Zhu et al., 2018). Indeed, our group and others have shown a modest chronic increase in plasma LPS levels in obesity, termed “metabolic endotoxemia” (Basu et al., 2011; Pendyala et al., 2012; Boutagy et al., 2016; Soto-Pantoja et al., 2021). It is imperative to mention that not all forms of endotoxemia are detrimental; LPS structural differences play a major role in host responses (Berezow et al., 2009; Vatanen et al., 2016). LPS structure is composed of three major moieties: lipid A, core oligosaccharide, and O-antigen (**Figure 4A**). Lipid A is the inner-most part that is responsible for the immunogenicity of LPS. It is an acylated and phosphorylated disaccharide of glucosamine that has varying length, number, distribution, and saturation of its fatty acid side chains. The general notion is that the immunogenicity of lipid A increases when the number of phosphate groups increases, the number of acyl chains, the number of acyl branching increases, or the “cumulative” lengths of the acyl chains decrease (**Figure 4**) (Coats et al., 2007; Anhê et al., 2021). The immunogenic forms of LPS are expressed by Proteobacteria (Coats et al., 2007; d’Hennezel et al., 2017) which have been consistently shown to be enriched in the gut microbiota of obese subjects (Xu et al., 2022).

**Figure 5:**
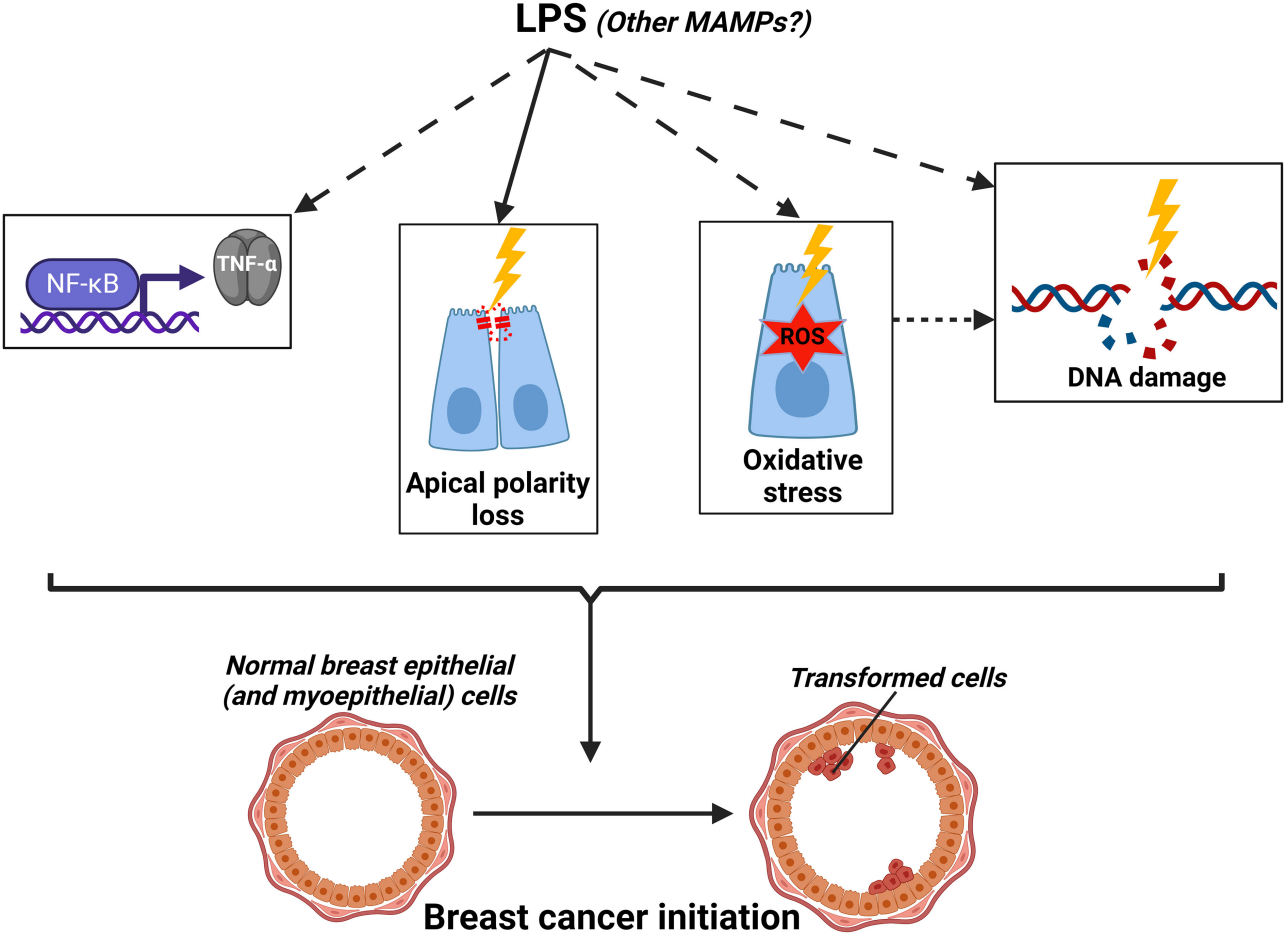
Working model by which LPS (and other metabolites) might cause breast cancer initiation (dashed arrows=hypothesized mechanisms yet to be demonstrated in the mammary gland context). Created with BioRender.com.

The luminal breast epithelium contains several cell-cell adhesion complexes such as the tight junctions (TJs) which segregate cell membrane components and receptors between the apical and basolateral domains, thus strictly defining apical polarity. Apical polarity is a functional biomarker of breast cancer risk. Loss of apical polarity is implicated in the expansion of the stem/progenitor pool, the activation of cell cycle signaling, and the mitotic spindle misalignment, which collectively lead to proliferation and multilayering of the epithelium; all key factors of tumorigenesis (Vidi et al., 2013). *In vitro*, apical polarity was compromised by LPS in 3D cultures of breast acini (Soto-Pantoja et al., 2021; Yassine et al., 2021). Moreover, the “obese microbiome” decreased the expression of the apical polarity marker zonula occludens-1 (ZO-1) in mice mammary glands (Soto-Pantoja et al., 2021). Besides the loss of apical polarity by LPS, genotoxicity could be a possible mechanism for obesity-associated breast carcinogenesis. HeLa cells infected with bacteria isolated from breast cancer patients (*E. coli* or *S. epidermidis*) showed increased levels of DNA double-stranded breaks (DSBs) (Urbaniak et al., 2016). Given the fact that *E. coli* and *S. epidermidis* are Gram-negative and Gram-positive bacteria, respectively, genotoxicity was likely induced by different MAMPs (not only LPS), or other microbiome-related processes. This DNA damage induction could be mediated through the activation of the NF-κB pathway which could, potentially, introduce DSBs upon translocation to the nucleus and transactivation of target genes (Le May et al., 2010; Le May et al., 2012). For instance, *H. pylori* was shown to recruit nucleotide excision repair (NER) endonucleases as a result of the NF-κB pathway activation in gastric cancer cells which led to the formation of DSBs (Hartung et al., 2015). Another possible mechanism for the genotoxicity is through the generation of reactive oxygen species (ROS) that can lead to the accumulation of nuclear oxidative stress. Hints could be drawn from the association of obesity in males with plasma LPS levels, sperm DNA oxidative stress (seminal 8-oxo guanine), and DNA damage (Pearce et al., 2019). This is also evident in Chlamydia infections which result in ROS generation, 8-oxo guanine formation, and impairment of the DNA damage response (Chumduri et al., 2013). ROS generation by MAMPs such as LPS, LTA, and flagellin is well-documented in many immune and epithelial cells (Hsieh et al., 2012; Kim et al., 2012; Burgueño et al., 2019; Fernández-Rojas et al., 2020; Cheng et al., 2022), however, this is yet to be confirmed in breast tissue/mammary gland context. The proposed working model by which LPS (and possibly other MAMPs) might cause breast cancer initiation is illustrated in **Figure 5**.

Experiments with transgenic mice also show that TLR4 signaling is involved in mammary carcinogenesis. The knockout (KO) of the receptors, co-receptor (CD14), or downstream effectors such as MYD88 decreased cancer cell growth *in vitro* and *in vivo*. Mammary epithelial cells with knocked-out TLR4 injected into cleared fat pads of WT recipient mice showed a decreased ability to repopulate the mammary glands in comparison to WT cells. Importantly, this decreased self-renewal capacity of TLR4 KO epithelial cells occurred in WT mice which indicated that this is an epithelial cell-intrinsic property independent of the immune microenvironment (Scheeren et al., 2014). Interestingly, immunostaining showed that LPS localizes in the cytoplasm and nuclei of breast tumor cells, and fluorescence *in situ* hybridization (FISH) against bacterial 16s rRNA revealed only a cytoplasmic signal (Nejman et al., 2020). This was corroborated in our animal study where mammary tumors from mice given lard diet showed strong cytoplasmic and nuclear LPS signals (Soto-Pantoja et al., 2021). LPS immunostaining in a spontaneous mammary tumor model showed a peri-nuclear punctate staining pattern with almost a complete absence of signal from the extracellular space (Fu et al., 2022). Because of the intracellular presence of LPS, this is suggestive of the involvement of TLR4-independent mechanisms such as direct genotoxicity or modulation of transcriptional activity.

The tumor-promoting effects of MAMPs could be indirectly affecting breast cancer through the creation of a suppressive immune microenvironment. Chronic exposure of macrophages to LPS switched their phenotype towards an M1 polarization which is pro-inflammatory. Breast cancer cells co-cultured with these macrophages (or treated with their conditioned medium) showed increased proliferation, motility, and clonogenicity (Roy et al., 2023). This view is corroborated by clinical evidence from women with pregravid obesity showing adipose tissue inflammation with increased accumulation of CD68+ M1 macrophages. These macrophages showed increased expression of LPS-sensing machinery such as TLR4 and CD14. This was attributed to the doubling of their plasma LPS levels in comparison to lean patients (Basu et al., 2011). These LPS-induced immunosuppressive effects are not limited to macrophages but may extend to T cells and other immune cells. Some hints could be drawn from a murine model of lung cancer where chronic exposure to LPS caused T-cell exhaustion and increased tumorigenesis. LPS-induced inflammation caused tumor accumulation of myeloid-derived suppressive cells and regulatory T cells and increased PD-1 expression. In this context, immune checkpoint blockade turned this immune-cold microenvironment hot and reduced tumorigenesis (Liu et al., 2021). Within breast cancer subtypes, TNBC showed the highest LPS accumulation and TLR4 expression in comparison to the other subtypes (Mehmeti et al., 2015; Feng et al., 2022). This goes well with the fact that TNBC subtype has the highest PD-1 expression which benefits the most from immune check blockade (Núñez Abad et al., 2022; Liu et al., 2023).

In addition to promoting breast tumorigenesis, accumulating evidence indicate that MAMPs promote breast cancer metastasis. TLR4 overexpression in breast tumors correlated with increased lymph node metastasis (Yang et al., 2014). TLR4 overexpression, specifically, by mononuclear inflammatory cells such as lymphocytes and monocytes was associated with an increased risk of metastasis in breast cancer patients (González-Reyes et al., 2010). *In vitro* assays showed that LPS treatments induced breast cancer cell invasion and migration. These effects were mediated by T-LAK cell-originated protein kinase (TOPK)-dependent enhancement of NF-κB transcriptional activity. In clinical samples, TLR4 and TOPK expression was significantly higher in high-grade breast cancer, invasive ductal carcinoma, and lymph node metastasis in comparison to low-grade samples and normal tissues (Seol et al., 2017). Other mechanisms were shown to mediate the LPS-induced breast cancer metastasis to other organs such as the prostaglandin E2-EP2 pathway (lung metastases) (Li et al., 2015) and the MYD88-leukotriene B_4_ receptor-2 axis (small bowel metastases) (Park and Kim, 2015).

##### 4.1.1.2 Flagellin

The effect of flagellin/TLR5 activation on breast cancer initiation, progression, and metastasis is much less studied (and contradictory at times) when compared to LPS/TLR4. A couple of studies showed that breast tumors overexpress TLR5 (Cai et al., 2011; Shuang et al., 2017). TLR5 expression also positively correlated with lymph node metastasis (Shuang et al., 2017) Yet, contradicting opposing associations were seen with tumor grade (Cai et al., 2011; Shuang et al., 2017). A nonsense single nucleotide polymorphism (SNP, rs5744168) that causes truncation of the transmembrane signaling domain of TLR5 was associated with higher breast cancer risk (Shuang et al., 2017). This goes along with a preceding study showing that patients with ER-positive breast cancers have lower overall survival when carrying the same mutated allele. It is also corroborated by the faster mammary tumor progression in TLR5 KO mice when compared to WT. This tumor-promoting activity was only preserved in mice with an intact microbiome and was lost when mice were treated with antibiotics. Mechanistically, the dysbiotic microbiome in TLR5 KO mice significantly increased IL-17 levels systemically and locally (in the tumor) which is believed to play a role in instigating tumor-promoting inflammation and dampened anti-tumor immunity. This increase in IL-17 levels was also seen in breast cancer patient samples with the TLR5 nonsense mutation. Relevant to this discussion, TLR5 KO mice showed opposite effects on ovarian tumors and sarcomas. Also, the TLR5 nonsense mutation did not show a significant association with survival in ovarian cancer patients. Noticeably, IL-6 (not IL-17) mediated the ovarian tumor-promoting effects in the WT mice by creating an immunosuppressive tumor microenvironment. It is compelling to determine why dysbiotic microbiomes in TLR5-deficient backgrounds lead to contrasting outcomes in breast cancer vs. ovarian cancers/sarcomas (Rutkowski et al., 2015).

*In vitro*, flagellin and TLR5 agonists decrease breast cancer cell proliferation, invasion, and migration (Cai et al., 2011; Shi et al., 2014). In addition, conditioned media from flagellin-treated breast cancer cells reduced proliferation which shows that soluble factors mediate an anti-proliferative autocrine communication between breast cancer cells (Cai et al., 2011). These direct effects (i.e., not mediated by immune cells) may be complementary to the indirect immune effects measured *in vivo*. *In vivo*, flagellin and TLR5 agonists decrease tumorigenesis by enhancing anti-tumor immunity (Cai et al., 2011; Gonzalez et al., 2023; Shakiba et al., 2023). Flagellin treatment notably increased neutrophil/lymphocyte infiltration into the tumors (Cai et al., 2011) and enhanced the efficacy of immune-checkpoint therapy (Gonzalez et al., 2023) and of oncolytic viral therapy (Shakiba et al., 2023). The effects of flagellin and TLR5 agonists on breast cancer initiation and metastasis are still unknown. An interesting aspect of flagellins is their varying degrees of TLR5-binding and stimulating capabilities. Three forms of flagellins have been proposed (Clasen et al., 2023): 1) the typical “stimulator” forms that have high binding and stimulating capabilities which are more prevalent in pathogens, 2) the “evader” forms that have low binding capabilities and, therefore, low stimulating capabilities, and 3) the “silent” forms that have high binding capability but, surprisingly, low stimulating capability which are more prevalent in commensals. It is unknown if flagellin levels and/or their different forms play a role in obesity-associated breast cancer risk.

##### 4.1.1.3 LTA

The literature on LTA’s presence in breast tumor tissues is contradictory with some groups reporting the absence of LTA staining (Nejman et al., 2020; Feng et al., 2022) and others, including our group, showing LTA signals in the majority of breast tumor tissues (Soto-Pantoja et al., 2021; Fu et al., 2022). It is more likely that there are Gram-positive bacteria residing in the tumor tissues with 16s rRNA sequencing analysis showing Gram-positive bacteria constituting at least 20-30% of the breast microbiome (Thompson et al., 2017; Klann et al., 2020; Nejman et al., 2020). While it is plausible that some bacteria lose their cell walls upon cellular internalization, this could not explain the absence of an LTA signal since this is a non-discriminant process that is not exclusive to Gram-positive bacteria (Errington, 2013; Nejman et al., 2020). LTA staining in a spontaneous mammary tumor model showed a peri-nuclear punctate pattern with almost a complete absence of signal from the extracellular space (Fu et al., 2022). Importantly, we documented the modulation of LTA levels in obesity where lard diet-fed mice showed strong cytoplasmic and nuclear signals compared to control diet-fed mice. We also showed in primary breast tumor samples that LTA positivity within epithelial cells strongly correlated with infiltrating CD45^+^ leukocytes (Soto-Pantoja et al., 2021).

Experiments with transgenic mice have shown that signaling of TLR2, the major pattern-recognition receptor for LTA, is involved in mammary carcinogenesis. The KO of the TLR2 receptor, co-receptor (CD14), or downstream effectors such as MYD88 decreased cancer cell growth *in vitro* and *in vivo*. Blockade of TLR2 with neutralizing antibodies decreased colony formation of breast cancer cells. Mammary epithelial cells with TLR2 KO injected into cleared fat pads had a decreased ability to repopulate the mammary glands in comparison to WT cells. Importantly, this decreased self-renewal capacity of TLR2 KO epithelial cells occurred in WT immuno-competent mice, indicating that this is an epithelial cell-intrinsic property independent of the immune microenvironment (Scheeren et al., 2014). Intra-tumoral depletion of LTA- and LPS-containing bacteria via antibiotics decreased lung metastasis of mammary tumors. However, if LTA plays a role in breast cancer metastasis (like LPS) is yet to be determined (Fu et al., 2022).

**Figure 6:**
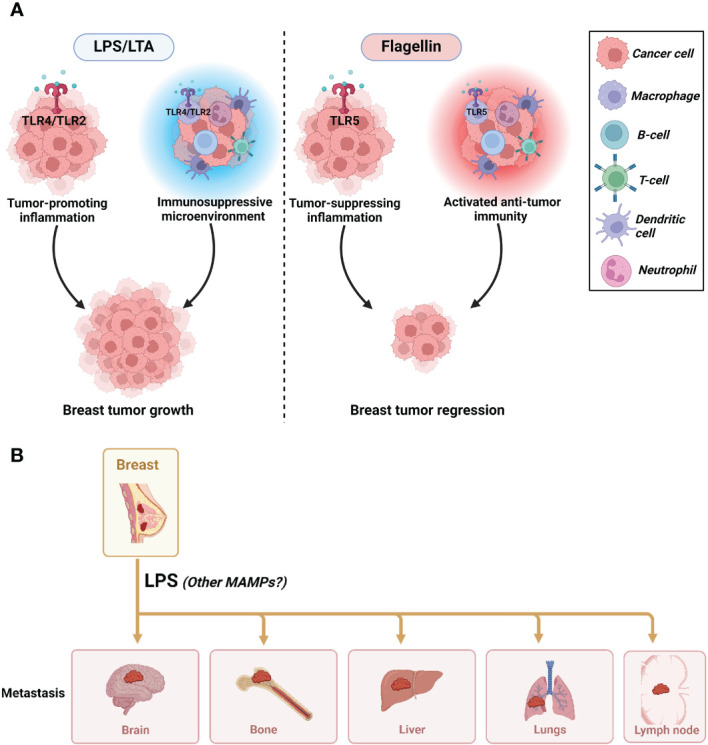
The impact of MAMPs on tumor progression and metastasis. **(A)** The mixed effects of MAMPs on breast cancer progression where LPS/LTA promote tumorigenesis and flagellin reduce tumorigenesis. **(B)** LPS promotes breast cancer metastasis. It is unknown if LTA and flagellin are implicated in promoting or inhibiting metastasis. Created with BioRender.com.

In conclusion, MAMPs such as LPS, LTA, and flagellin could be a case of mixed blessings in breast cancer (**Figure 6**). One MAMP could be beneficial or detrimental depending on the stage in breast carcinogenesis (initiation vs. progression vs. metastasis), the different subtypes of breast cancer, the different forms of these MAMPs (immunogenic vs. non-immunogenic forms), and the different host-related factors (diet, BMI, genetics). This underscores the profound complexity of studying the obesity-driven modulation of MAMPs in breast cancer.

#### 4.1.2 Short chain fatty acids

The gut microbiome is the main source of SCFA, which are one- to six-carbon-length saturated aliphatic organic acids. While the host can synthesize a small amount of some SCFA through biological processes, the bacterial microbiome produces 90% of SCFA by fermentation of dietary fibers (Bourlioux et al., 2003). These microbes mainly produce acetate (C2), propionate (C3), and butyrate (C4) which are the major SCFAs produced mainly in the proximal colon at high concentrations (70 - 140 mM) (Tan et al., 2014). Multiple molecular signaling functions are attributed to SCFAs (Xiong et al., 2022), including immunomodulatory effects via *i)* ROS production, chemotaxis, and phagocytosis, *ii)* stimulation of gut motility and nutrient absorption, *iii)* anti-microbial, *iv)* anti-inflammatory, and *v)* anti-tumorigenic properties. SCFAs elicit physiological effects through the inhibition of histone deacetylases (HDACs) and the activation of G-protein-coupled receptors (GPCRs) such as GPR43, GPR41, and GPR109A (Boulangé et al., 2016; Parada Venegas et al., 2019). Of particular importance is the activation of GPR109A by SCFAs which suppresses NF-κB activation and the subsequent production of pro-inflammatory cytokines (Thangaraju et al., 2009; Tan et al., 2014). In addition, SCFAs induce lipolysis via binding to GPR43 which leads to the release of free fatty acids that can bind to TLRs and activate pro-inflammatory pathways (Jia et al., 2017; Picon-Ruiz et al., 2017). This is also accompanied by a reduction in the levels of circulating leptin; an adipokine that is known to cause apical polarity loss in 3D cultures of breast acini and *in vivo* (Tenvooren et al., 2019). Moreover, the global inhibition of HDACs by SCFAs correlates with increased acetylation of histones and decreased cytokine production (Tan et al., 2014; Parada Venegas et al., 2019); the modulation of the NF-κB pathway by HDAC inhibition is one plausible mechanism between SCFAs and reduced breast carcinogenesis.

The amount of SCFAs depends on various host, environmental, dietary, and gut microbiota factors. In rodents, ovariectomy reduced SCFA metabolite bioavailability, which was partially restored with *Lactobacillus* probiotic or high-fiber diet administration (Chen et al., 2021). Clinical studies have also determined that aging and menopause are associated with decreased SCFA (Kirschner et al., 2023). A cross-sectional study investigating both the plasma and fecal SCFA showed that circulating (but not fecal) butyrate and propionate (but not acetate) were inversely related to BMI (Muller et al., 2019). These data indicate that both menopause and obesity are associated with decreased plasma SCFA.

Butyrate is mostly produced by bacteria from the *Firmicutes* phylum such as *Clostridium leptum* and *Faecalibacterium prausnitzii.* Butyrate is also produced from acetate or lactate precursors by sugar-and/or lactate-utilizing bacteria such as *Eubacterium hallii* and *Anaerostipes* spp. Propionate and acetate are produced by the mucin-degrading bacteria *Akkermansia muciniphila*. Acetate also is produced during carbohydrate fermentation by many *Bifidobacterium* species (Parada Venegas et al., 2019). Higher body fat in breast cancer patients was associated with lower gut abundance of SCFA-producing *Akkermansia muciniphila*, lower alpha diversity, and higher levels of the pro-inflammatory cytokine IL-6 (Fruge et al., 2020). A study showed a reduction in many SCFA-producing bacteria, including *Faecalibacterium* prausnitzii, *Parabacteroides merdae*, and *Alistipes*, in breast cancer patients compared to healthy controls. Functional analysis showed a marked decrease in propionate production in breast cancer patients (Shrode et al., 2023). In a different study (Zhu et al., 2018), the butyrate-producing bacteria *Roseburia inulinivorans* were significantly less abundant in postmenopausal breast cancer patients compared to controls. Gene set enrichment analysis confirmed the lower expression of butyrate synthesis genes in breast cancer patients. Finally, the butyrate-producing bacteria *Faecalibacterium prausnitzii* was less abundant in obese/overweight breast cancer patients than in patients with normal BMI (Luu et al., 2017; Ma et al., 2020). This is in tandem with clinical studies of infectious colitis and inflammatory bowel disease showing an association between decreased *Faecalibacterium prausnitzii* abundance, decreased butyrate production, and increased inflammation (Martinez-Medina et al., 2006; Sokol et al., 2008). All-in-all, this supports the notion that the reduction in SCFA-producing bacteria, and hence SCFAs, is a major contributor to the increased breast tumorigenesis in obesity.

### 4.2 Insulin signaling

Dysbiosis in obesity influences the development of insulin resistance, a condition associated with increased breast cancer incidence and mortality (Lauby-Secretan et al., 2016; Marzullo et al., 2021). Germ-free mice did not develop insulin resistance on a high-fat diet, contrary to animals with an intact gut microbiome (Bäckhed et al., 2004). Fecal transplantation of an “obese microbiome” from *ob*/*ob* mice to germ-free mice caused a higher increase in fat storage and energy harvest than a FT of a lean mice microbiome, and led to insulin resistance (Turnbaugh et al., 2006). Several factors contribute to insulin resistance in obesity such as reduced production of SCFAs and increased production of both bile acids and branched-chain amino acids (Saad et al., 2016). Increased LPS/TLR4 signaling (i.e., metabolic endotoxemia) is another factor: lean mice developed insulin resistance and glucose intolerance after chronic LPS infusions (Cani et al., 2007; Cani et al., 2008). TLR4 inhibition, loss-of-function mutation, or TLR4 KO prevents the development of insulin resistance, implicating this receptor in disease development (Poggi et al., 2007; Shinozaki et al., 2011; Saad et al., 2016).

Preclinical studies have identified a role for insulin in stimulating mammary tumor growth (Lohmann et al., 2016). Furthermore, prospective observational studies have identified positive associations between insulin levels and breast cancer incidence (Gunter et al., 2009; Gunter et al., 2015). A study by Pan et al. investigating the associations between insulin resistance and breast cancer incidence in postmenopausal women found higher levels of insulin resistance are associated with higher breast cancer incidence and higher all-cause mortality after breast cancer (Pan et al., 2020). A recent study demonstrated an accumulation of microbiome-derived metabolites in breast tumors from obese diabetic women that was associated with DNA damage repair deficiency (Panigrahi et al., 2023). These metabolites included imidazole propionate, phenyl sulfate, and trimethylamine N-oxide which were shown previously to induce the generation of ROS and increase inflammation (Kikuchi et al., 2019; Molinaro et al., 2020; Lemaitre et al., 2021). As components of the metabolic syndrome, the link between obesity and the development of insulin resistance is strong. Yet, further research is required to determine if dysbiosis associated with obesity plays a role in the development of breast cancer through insulin resistance.

### 4.3 Estrogen bioavailability

There are three main forms of endogenous estrogens: estrone (E1), estradiol (E2), and estriol (E3) which are the dominant forms during post-menopause, pre-menopause, and pregnancy, respectively (Plottel and Blaser, 2011). According to the International Agency for Research on Cancer (IARC) latest monographs (I.A.R.C., 2023), estrogen is carcinogenic in the breast (among other sites). Estrogen drives breast cancer development through mutagenesis, proliferation, angiogenesis, and ultimately metastasis through estrogen receptor-dependent and independent mechanisms (Clusan et al., 2023). The centrality of estrogen in breast cancer is reflected by the wide usage of breast cancer therapeutics targeting this hormone or its receptors, such as selective estrogen receptor modulators (SERMs), selective estrogen receptor degraders (SERDs), and aromatase inhibitors (AIs) (Lumachi et al., 2015; Burstein et al., 2021). Estrogens undergo Phase I oxidative metabolism and Phase II conjugation reactions in the liver. First, they undergo irreversible hydroxylation via NADPH-dependent cytochrome P450 (CYP) enzymes to form catechol estrogens. Then, Phase II reactions include glucuronidation via uridine 5’-diphospho-glucuronosyltransferase, methylation via catechol-O-methyltransferase, and sulfonation via sulphotransferase (Raftogianis et al., 2000). Glucuronidated estrogens are hydrophilic and generally more polar than parent estrogens, which allows them to dissolve in blood and get excreted in urine. However, studies have found that considerable amounts of estrogens enter the gastrointestinal tract via biliary secretion for further metabolism (Parida and Sharma, 2019). Here, gut microbial β-glucuronidase (GUS) enzymes may deconjugate glucuronidated estrogens, releasing the parent estrogen aglycones, which are rendered available for reabsorption. Thus, the gut microbiome establishes the enterohepatic recirculation of estrogens, increasing their bioavailability. This explains the increase in the fecal excretion of conjugated estrogens in humans treated with antibiotics and the concomitant decrease in urinary levels. This also shows the important role of the gut microbiome in estrogen homeostasis (Ervin et al., 2019).

Associations between gut microbiome composition/diversity and estrogen/GUS levels have been demonstrated in multiple studies. Fuhrman et al. (2014) found that a higher diversity of the gut microbiome is associated with a higher ratio of urinary estrogen metabolites to parent estrogens. Additionally, Flores et al. (2012) showed an association between microbial alpha diversity and the levels of urinary estrogens and estrogen metabolites. Urinary estrogens were associated with the abundance of several *Clostridia* taxa that express GUS enzymes (*non-Clostridiales* and three genera in the *Ruminococcaceae* family). Urinary estrone levels were associated with fecal GUS levels. In contrast, fecal estrogen levels were inversely associated with GUS levels. This shows the effect of GUS on increasing systemic estrogen levels by increasing their reabsorption from the gut and contributing to the increased breast cancer risk.

Specific gut bacteria have been linked to estrogen metabolism. For example, the abundance of the GUS-producing bacteria *Erwinia amylovora* correlated with estradiol levels (Zhu et al., 2018). Interestingly, this bacterium was enriched in the gut microbiome of breast cancer patients compared to cancer-free women. The abundance of other GUS-producing bacteria (*Clostridium leptum*, *Clostridium coccoides*, and *Faecalibacterium prausnitzii*) was associated with higher tumor stages (Luu et al., 2017). More importantly, obesity and obesogenic diets have been shown to modulate GUS-producing bacteria (Arnone and Cook, 2022). High fat diet-fed mice showed an increased GUS activity in comparison to low fat diet-fed mice (Creekmore et al., 2019). Omnivorous women showed higher fecal GUS activity, lower fecal estrogen excretion, and higher plasma estrone and estradiol levels in comparison to vegetarians (Goldin et al., 1982). In summary, elevated levels of circulating estrogens are a hallmark of adiposity and GUS-producing bacteria contribute to this obesity-mediated increase in estrogen bioavailability.

### 4.4 Microbial-derived metabolites as signaling molecules

Changes in the gut microbiome composition lead to changes in the microbiome metabolome which, in turn, may act as a mediator of carcinogenesis in distant tissue sites. Altered microbial bile acid (BA) metabolism is a hallmark of both obesity and breast cancer (Di Ciaula et al., 2017). In addition to cholesterol and phospholipids, bile acids are one of the three lipid components of biliary secretion (bile). Primary bile acids such as cholic acid and chenodeoxycholic acid are synthesized in the liver from cholesterol by the CYP enzymes. Then, primary bile acids are conjugated to taurine and glycine, which renders them more hydrophilic and ready for secretion (Long et al., 2017). The gut microbiome deconjugates primary bile acids and causes their further biotransformation into secondary and tertiary bile acids. Cholic acid and chenodeoxycholic acid are the precursors of the secondary bile acids, deoxycholic acid (DCA) and lithocholic acid, respectively. Epimerization of lithocholic acid leads to the formation of the tertiary bile acid, ursodeoxycholic acid (Ridlon et al., 2006). The general perception is the higher the hydrophobicity of a bile acid, the higher is its cytotoxicity. The hydrophobicity of bile acids is in the following order: lithocholic acid > DCA > chenodeoxycholic acid > cholic acid > ursodeoxycholic acid (Di Ciaula et al., 2017). In addition to the physiological roles of bile acids in the solubilization and absorption of dietary lipids and fat-soluble vitamins, they act as signaling molecules by activating specific nuclear receptors such as farnesoid X receptor (FXR), pregnane X receptor (PXR), and vitamin D receptor (VDR). Moreover, they activate membrane GPCRs such as the G-protein-coupled bile acid receptor-1 (GPBAR-1, *aka* TGR5), as well as downstream signaling pathways such as ERK and JNK (Martinot et al., 2017). Bile acid interactions with these receptors aid in the regulation of cellular energetics and nutrient metabolism of glucose, lipid and lipoprotein (Li and Chiang, 2014; Zhou and Hylemon, 2014; Di Ciaula et al., 2017).

Obesity is associated with aberrant regulation of BAs, whereby obesity alters BA composition, resulting in increased DCA and decreased cholic acid (Di Ciaula et al., 2017; Chávez-Talavera et al., 2019). BA composition alterations in obesity were shown to be mediated by the gut microbiome which, in turn, caused alterations in BA signaling and host metabolism (Wei et al., 2020). Obesity leads to reduced postprandial BA release and increased levels of 12-alpha-hydroxylated BA forms, which are elevated in individuals with insulin resistance by as much as twofold compared to healthy controls (Haeusler et al., 2013). DCA, a 12-alpha-hydroxylated secondary BA, has been shown to act as a tumor promoter by decreasing apoptosis in breast cancer progenitor cells (Krishnamurthy et al., 2008). Moreover, DCA concentrations are 50 times higher in human breast cyst fluid than plasma concentrations (Javitt et al., 1994). A case–control study comparing postmenopausal breast cancer patients with age- and BMI-matched healthy controls found mean plasma DCA concentration to be 52% higher in the breast cancer patients (Costarelli and Sanders, 2002).

Several lines of evidence show an inverse association between bile acids or their receptors and breast cancer. Miko et al. (2018), showed lower serum levels of lithocholic acid and reduced ratio of chenodeoxycholic acid to lithocholic acid in breast cancer patients than healthy controls. Moreover, breast cancer patients had a reduced abundance of the 7α/β-hydroxysteroid dehydroxylase gene (coding for a key enzyme in lithocholic acid generation) in their fecal DNA. Low lithocholic acid levels induce lipogenesis by upregulating lipid synthesizing enzymes (SREBP-1c, FASN, and ACACA), as well as proliferation by decreasing the expression of pro-apoptotic proteins (Bax and Bcl-2). In a study by Giaginis et al. (2017) on invasive breast carcinoma, low expression of the lithocholic acid receptor (FXR) was associated with larger tumor sizes, higher Ki67 expression, and shorter overall and disease-free survival. Tang et al. (2019) reported similar findings, with higher chenodeoxycholic acid and DCA levels in breast tumors than in normal tissues. Again, the bile acid precursors were inversely correlated with the expression of cell cycle regulators and cell proliferation in breast tumors. In summary, increased breast tumorigenesis could be mediated by BA alterations in obesity such as increased DCA, increased chenodeoxycholic acid, and decreased lithocholic acid.

Other bacterial metabolites, in particular tryptophan derivatives, are relevant to breast cancer. Tryptophanase A is responsible for the deamination of tryptophan into the cytostatic metabolite; indolepropionic acid. Fecal samples from breast cancer patients showed lower bacterial tryptophanase A gene abundance in comparison to cancer-free controls; which is indicative of lower bacterial indolepropionic acid biosynthesis in breast cancer patients. Moreover, tryptophanase A gene abundance positively correlated with the number of tumor-infiltrating lymphocytes, which partly explains the lower anti-tumor immunity in breast cancer patients (Sári et al., 2020a). Reduced levels of two bacterial tryptophan metabolites (indolepropionic acid and indoxylsulfate) are associated with increased breast tumorigenesis. At the tumor level, lower expression of tryptophan metabolite receptors (aryl hydrocarbon receptor; AHR) and PXR was associated with lower survival in breast cancer patients. *In vitro*, increasing concentrations of indolepropionic acid and indoxylsulfate reduced stemness, proliferation, and epithelial-to-mesenchymal transition (EMT) of breast cancer cells (Sári et al., 2020a; Sári et al., 2020b). Finally, bacterial metabolism transforms tryptophan into indole which is then hydroxylated by Cyp2e1 and sulfated by SULT1 and SULT2 enzymes in the liver to produce indoxylsulfate. Reduced expression of these liver enzymes was associated with lower survival in breast cancer patients. Tryptophan metabolism has also been associated with obesity and TNBC. (Smith A, et al., 2022) investigated alterations in microbial metabolism pathways in breast tissues of obese women relative to non-obese women with and without TNBC. Random forest analysis showed a unique biochemical signature associated with elevated L-Tryptophan and Kynurenine metabolites and lower levels of microbial-derived metabolites critical for controlling inflammation and immune response in obese individuals and those with TNBC. Additionally, analysis of The Cancer Genome Atlas revealed that the expression of key L-Tryptophan enzymes was significantly associated with worse survival outcomes in TNBC patients (Smith et al., 2022). Overall, these findings suggest a complex interplay between bacterial metabolism, tryptophan derivatives, obesity, and breast cancer development and progression. Further research is needed to fully understand the mechanisms underlying these associations.

Another metabolite, cadaverine, is produced from its lysine precursor via the lysine decarboxylase enzymes which are expressed by numerous bacterial species. Breast cancer patients had a reduced abundance of bacterial lysine decarboxylase genes in their fecal samples than healthy controls, which indicated lower bacterial cadaverine production. Moreover, lower expression of lysine decarboxylases was associated with shorter survival in breast cancer patients (Kovacs et al., 2019). The levels of cadaverine has been shown to positively correlate with BMI in non-cancer subjects (Loftfield et al., 2020). This shows that cadaverine might act as a two-edged sword where its cytotoxicity could be desired for breast tumor regression yet detrimental for healthy breast epithelial cells.

Bacterial-derived toxins are also implicated in breast carcinogenesis. *Bacteroidetes fragilis* is an important gut commensal but can function as a potent pathogen through the production of *Bacteroides fragilis* toxin (BFT). Enteric abundance of *B. fragilis* is also strongly linked with obesity. *B. fragilis* is thought to accelerate obesity by suppressing acetic acid levels (Shen et al., 2022). Enterotoxigenic *B. fragilis* (ETBF) is capable of inducing oncogenic transformation in the gut mucosa, leading to the formation of spontaneous tumors (Sears et al., 2014). ETBF infection also aids in the establishment of the premetastatic niche through increased proinflammatory and protumorigenic cytokines. The bacteria also induces remodeling of the tumor microenvironment via immune cell and stroma infiltration (Parida et al., 2023). Furthermore, the toxin-producing strains of *Bacteroides fragilis* are known for inducing colitis and colon neoplasia in mice. Parida et al. recently demonstrated the effect of BFT on mammary tumorigenesis (Parida et al., 2021). Colonization of the mammary glands and the gut with enterotoxigenic *B. fragilis* caused hyperplasia in the mammary glands. It also increased tumorigenesis and metastasis in mice to a greater extent than the nontoxigenic strains. Notch1 and β-catenin signaling axes were identified as mediators of the BFT carcinogenesis process. Taken together, these findings emphasize the complex role obesity-specific bacterial species and their toxins can play in promoting breast cancer development and progression.

## 5 Effects of obesity on the breast microbiome

In 2014 Urbaniak et al. (2014) established the existence of a breast microbiome and inspired more than a dozen studies demonstrating dysbiosis in breast cancer that are well-summarized in ref (Peters et al., 2023). Differences in the breast microbiome were also found between breast cancer patients and patients with non-malignant breast disease (Hieken et al., 2016; Urbaniak et al., 2016), between breast tumors and paired tumor-adjacent normal tissue (Thompson et al., 2017; Smith et al., 2019; Esposito et al., 2022), and between breast cancer survivors and women who never had breast cancer (Chan et al., 2016; Klann et al., 2020). An interesting comparison of beta diversity was done by Costantini et al. (2018) between three groups: 1) paired tumors and tumor-adjacent normal tissues, 2) tumors of different subjects and 3) tumor-adjacent normal tissues of different subjects. The beta diversity was significantly lower in paired tumors and normal-adjacent tissues within the same subject than in tumors or normal-adjacent tissues of different subjects. This shows that more similarities than differences exist in the microbiome of tumors and normal-adjacent tissues within individuals. Hence, it may indicate that dysbiosis in the breast is antecedent to tumor initiation by establishing a pro-tumorigenic microenvironment.

Obesity induces microbiome perturbations in the breast tissue. Our group has demonstrated that obesity modifies tumoral microbiome populations in the breast (Chiba et al., 2020). We have also shown that an obesogenic Western diet perturbs the breast microbiome in non-human primates. Obesogenic Western diet disturbed non-cancerous breast tissue homeostasis by significantly decreasing bile acid levels and increasing oxidative stress; mechanisms that are associated with increased breast cancer risk (Shively et al., 2018). More studies are needed in the breast cancer initiation front to identify the pre-malignant changes that are likely to occur in obesity due to breast microbiome perturbations.

## 6 Conclusions, challenges and future perspectives in gut microbiome research

In conclusion, obesity modulates the gut microbiome in ways that may increase breast cancer risk. Carcinogenic mediators communicate gut microbiome changes in obesity to the breast. These mediators include circulating LPS, SCFAs, estrogens, IGF-1, and DCA which can influence molecular signaling at distant tissue sites such as the breast. However, studying the impact of these mediators on breast cancer risk needs to be carried in the context of obesity to prove causality.

A major challenge in gut microbiome research is the lack of reproducibility between studies. Although some findings align well, there are many differences and contradictions (**Table 1**). For instance, compositional differences in the gut microbiome between premenopausal breast cancer patients and healthy controls were reported by He et al. (2021), while no differences were found by Zhu et al. (2018). Although both study populations were Chinese; the methodologies used for microbiome identification and inclusion criteria were different. The former used 16s rRNA sequencing while the latter used metagenomic sequencing. Inclusion criteria were also different; the former study excluded patients exposed to antibiotics within one month of fecal sample collection while the latter had a wider exclusion window of three months. Another example is the increase in F/B ratio in breast cancer patients *vs*. healthy controls observed by Bobin-Dubigeon et al. (2021) but not by Byrd et al. (2021). The discrepancy could be explained by the different populations studied (European vs. African) and the methodologies used. The former study used RT-qPCR to quantify bacteria copy numbers, while the latter used 16s rRNA sequencing.

Overall, several factors may (at least partly) explain discrepancies between gut microbiome studies, including differences in study population (geography, BMI, race/ethnicity, diet, xenobiotic exposure, inclusion/exclusion criteria), sample handling (collection method, storage time, preservatives, external contaminants), experimental protocols (DNA extraction, library preparation, sequencing methodology, reference database), bioinformatics pipelines, and statistical analyses. How variability in these factors leads to different findings and potential solutions for them are comprehensively illustrated in this review (Nearing et al., 2021). Ideally, a universal standardized protocol for microbiome studies should be adopted to eliminate many of the aforementioned variability.

Future research on the interactions between obesity and the gut microbiome on breast cancer development is needed. A better understanding is needed of how an individual’s gut microbiome is influenced by a combination of factors such as obesity, diet, and genetics and how such combinations affect treatment responses. This could lead to personalized treatment strategies that consider the patient’s microbiome. Additionally, further investigation into microbial metabolites specific for obesity, immune system interactions, and hormonal pathways that play a role in breast cancer development and progression are needed. Finally, the determination of obese microbial markers or signatures that can be used for the identification of high-risk individuals and early detection of breast cancer will aid the development of prevention strategies and early interventions. Advancements in these areas will have a positive impact on the breast cancer incidence and mortality of obese populations.

## Author contributions

MG: Writing – original draft. AA: Writing – original draft. P-AV: Writing – review & editing. KC: Writing – review & editing.

## References

[B1] Aarnoutse R. Hillege L. E. Ziemons J. De Vos-Geelen J. de Boer M. Aerts E.. (2021). Intestinal microbiota in postmenopausal breast cancer patients and controls. Cancers (Basel) 13. doi: 10.3390/cancers13246200 PMC8699039 34944820

[B2] Anhê F. F. Barra N. G. Cavallari J. F. Henriksbo B. D. Schertzer J. D.. (2021). Metabolic endotoxemia is dictated by the type of lipopolysaccharide. Cell Rep. 36, 109691. doi: 10.1016/j.celrep.2021.109691 34525353

[B3] Argolo D. F. Hudis C. A. Iyengar N. M. (2018). The impact of obesity on breast cancer. Curr. Oncol. Rep. 20, 47. doi: 10.1007/s11912-018-0688-8 29644507

[B4] Arnone A. A. Cook K. L. (2022). Gut and breast microbiota as endocrine regulators of hormone receptor-positive breast cancer risk and therapy response. Endocrinology 164. doi: 10.1210/endocr/bqac177 PMC9923803 36282876

[B5] Avgerinos K. I. Spyrou N. Mantzoros C. S. Dalamaga M. (2019). Obesity and cancer risk: Emerging biological mechanisms and perspectives. Metabolism 92, 121–135. doi: 10.1016/j.metabol.2018.11.001 30445141

[B6] Bäckhed F. Ding H. Wang T. Hooper L. V. Koh G. Y. Nagy A.. (2004). The gut microbiota as an environmental factor that regulates fat storage. Proc. Natl. Acad. Sci. U.S.A. 101, 15718–15723. doi: 10.1073/pnas.0407076101 15505215 PMC524219

[B7] Bäckhed F. Manchester J. K. Semenkovich C. F. Gordon J. I. (2007). Mechanisms underlying the resistance to diet-induced obesity in germ-free mice. Proc. Natl. Acad. Sci. U.S.A. 104, 979–984. doi: 10.1073/pnas.0605374104 17210919 PMC1764762

[B8] Basu S. Haghiac M. Surace P. Challier J. C. Guerre-Millo M. Singh K.. (2011). Pregravid obesity associates with increased maternal endotoxemia and metabolic inflammation. Obes. (Silver Spring) 19, 476–482. doi: 10.1038/oby.2010.215 PMC3628602 20930711

[B9] Bawaneh A. Wilson A. S. Levi N. Howard-McNatt M. M. Chiba A. Soto-Pantoja D. R.. (2022). Intestinal microbiota influence doxorubicin responsiveness in triple-negative breast cancer. Cancers (Basel) 14. doi: 10.3390/cancers14194849 PMC9563306 36230772

[B10] Berezow A. B. Ernst R. K. Coats S. R. Braham P. H. Karimi-Naser L. M. Darveau R. P. (2009). The structurally similar, penta-acylated lipopolysaccharides of Porphyromonas gingivalis and Bacteroides elicit strikingly different innate immune responses. Microb. Pathog. 47, 68–77. doi: 10.1016/j.micpath.2009.04.015 19460428 PMC2707506

[B11] Bertazzoni E. Beghini A. Vesentini S. Marchiori L. Nardo G. Cerutti R.. (2006). Intestinal microflora as an alternative metabolic source of estrogens in women with uterine leiomyoma and breast cancer. Ann. New York Acad. Sci. 595, 473–479. doi: 10.1111/j.1749-6632.1990.tb34337.x

[B12] Bisanz J. E. Upadhyay V. Turnbaugh J. A. Ly K. Turnbaugh P. J. (2019). Meta-analysis reveals reproducible gut microbiome alterations in response to a high-fat diet. Cell Host Microbe 26, 265–272.e4. doi: 10.1016/j.chom.2019.06.013 31324413 PMC6708278

[B13] Bobin-Dubigeon C. Luu H. T. Leuillet S. Lavergne S. N. Carton T. Le Vacon F.. (2021). Faecal microbiota composition varies between patients with breast cancer and healthy women: A comparative case-control study. Nutrients 13. doi: 10.3390/nu13082705 PMC8399700 34444865

[B14] Boulangé C. L. Neves A.L. Chilloux J. Nicholson J. K. Dumas M. -E. (2016). Impact of the gut microbiota on inflammation, obesity, and metabolic disease. Genome Med. 8, 42. doi: 10.1186/s13073-016-0303-2 27098727 PMC4839080

[B15] Bourlioux P. Koletzko B. Guarner F. Braesco V. (2003). The intestine and its microflora are partners for the protection of the host: report on the Danone Symposium “The Intelligent Intestine,” held in Paris, June 14, 2002. Am. J. Clin. Nutr. 78, 675–683. doi: 10.1093/ajcn/78.4.675 14522724

[B16] Boutagy N. E. McMillan R. P. Frisard M. I. Hulver M. W. (2016). Metabolic endotoxemia with obesity: Is it real and is it relevant? Biochimie 124, 11–20. doi: 10.1016/j.biochi.2015.06.020 26133659 PMC4695328

[B17] Brown K. A. (2021). Metabolic pathways in obesity-related breast cancer. Nat. Rev. Endocrinol. 17, 350–363. doi: 10.1038/s41574-021-00487-0 33927368 PMC10410950

[B18] Burgueño J. F. Fritsch J. Santander A. M. Brito N. Fernández I. Pignac-Kobinger J.. (2019). Intestinal epithelial cells respond to chronic inflammation and dysbiosis by synthesizing H(2)O(2). Front. Physiol. 10, 1484. doi: 10.3389/fphys.2019.01484 31871440 PMC6921703

[B19] Burstein H. J. Curigliano G. Thürlimann B. Weber W. P. Poortmans P. Regan M. M.. (2021). Customizing local and systemic therapies for women with early breast cancer: the St. Gallen International Consensus Guidelines for treatment of early breast cancer 2021. Ann. Oncol. 32, 1216–1235. doi: 10.1016/j.annonc.2021.06.023 34242744 PMC9906308

[B20] Byrd D. A. Vogtmann E. Wu Z. Han Y. Wan Y. Clegg-Lamptey J. N.. (2021). Associations of fecal microbial profiles with breast cancer and nonmalignant breast disease in the Ghana Breast Health Study. Int. J. Cancer 148, 2712–2723. doi: 10.1002/ijc.33473 33460452 PMC8386185

[B21] Cai Z. Sanchez A. Shi Z. Zhang T. Liu M. Zhang D. (2011). Activation of Toll-like receptor 5 on breast cancer cells by flagellin suppresses cell proliferation and tumor growth. Cancer Res. 71, 2466–2475. doi: 10.1158/0008-5472.CAN-10-1993 21427357 PMC3074302

[B22] Cani P. D. Amar J. Iglesias M. A. Poggi M. Knauf C. Bastelica D.. (2007). Metabolic endotoxemia initiates obesity and insulin resistance. Diabetes 56, 1761–1772. doi: 10.2337/db06-1491 17456850

[B23] Cani P. D. Bibiloni R. Knauf C. Waget A. Neyrinck A. M. Delzenne N. M.. (2008). Changes in gut microbiota control metabolic endotoxemia-induced inflammation in high-fat diet-induced obesity and diabetes in mice. Diabetes 57, 1470–1481. doi: 10.2337/db07-1403 18305141

[B24] Carding S. Verbeke K. Vipond D. T. Corfe B. M. Owen L. J. (2015). Dysbiosis of the gut microbiota in disease. Microb. Ecol. Health Dis. 26, 26191. doi: 10.3402/mehd.v26.26191 25651997 PMC4315779

[B25] Chan A. A. Bashir M. Rivas M. N. Duvall K. Sieling P. A. Pieber T. R.. (2016). Characterization of the microbiome of nipple aspirate fluid of breast cancer survivors. Sci. Rep. 6, 28061. doi: 10.1038/srep28061 27324944 PMC4914981

[B26] Chan D. S. M. Abar L. Cariolou M. Nanu N. Greenwood D. C. Bandera E. V.. (2019). World Cancer Research Fund International: Continuous Update Project-systematic literature review and meta-analysis of observational cohort studies on physical activity, sedentary behavior, adiposity, and weight change and breast cancer risk. Cancer Causes Control 30, 1183–1200. doi: 10.1007/s10552-019-01223-w 31471762

[B27] Chávez-Talavera O. Haas J. Grzych G. Tailleux A. Staels B. (2019). Bile acid alterations in nonalcoholic fatty liver disease, obesity, insulin resistance and type 2 diabetes: what do the human studies tell? Curr. Opin. Lipidol. 30, 244–254. doi: 10.1097/MOL.0000000000000597 30893108

[B28] Chen Q. Wang B. Wang S. Qian X. Li X. Zhao J.. (2021). Modulation of the gut microbiota structure with probiotics and isoflavone alleviates metabolic disorder in ovariectomized mice. Nutrients 13. doi: 10.3390/nu13061793 PMC8225012 34070274

[B29] Cheng C. Y. Chen Y. H. Thuy Tien Vo T. Chui Hong Y. Wang C. S. Canh Vo Q.. (2022). CORM-2 prevents human gingival fibroblasts from lipoteichoic acid-induced VCAM-1 and ICAM-1 expression by inhibiting TLR2/MyD88/TRAF6/PI3K/Akt/ROS/NF-κB signaling pathway. Biochem. Pharmacol. 201, 115099. doi: 10.1016/j.bcp.2022.115099 35617999

[B30] Chiba A. Bawaneh A. Velazquez C. Clear K. Y. J. Wilson A. S. Howard-McNatt M.. (2020). Neoadjuvant chemotherapy shifts breast tumor microbiota populations to regulate drug responsiveness and the development of metastasis. Mol. Cancer Res. 18, 130–139. doi: 10.1158/1541-7786.MCR-19-0451 31628201 PMC9153322

[B31] Chumduri C. Gurumurthy Rajendra K. Zadora Piotr K. Mi Y. Meyer Thomas F. (2013). Chlamydia infection promotes host DNA damage and proliferation but impairs the DNA damage response. Cell Host Microbe 13, 746–758. doi: 10.1016/j.chom.2013.05.010 23768498

[B32] Clasen S. J. Bell M. E.W. Borbón A. Lee D. H. Henseler Z. M. de la Cuesta-Zuluaga J.. (2023). Silent recognition of flagellins from human gut commensal bacteria by Toll-like receptor 5. Sci. Immunol. 8, eabq7001. doi: 10.1126/sciimmunol.abq7001 36608151

[B33] Clusan L. Ferrière F. Flouriot G. Pakdel F. (2023). A basic review on estrogen receptor signaling pathways in breast cancer. Int. J. Mol. Sci. 24. doi: 10.3390/ijms24076834 PMC10095386 37047814

[B34] Coats S. R. Do C. T. Karimi-Naser L. M. Braham P. H. Darveau R. P. (2007). Antagonistic lipopolysaccharides block E. coli lipopolysaccharide function at human TLR4 via interaction with the human MD-2 lipopolysaccharide binding site. Cell Microbiol. 9, 1191–1202. doi: 10.1111/cmi.2007.9.issue-5 17217428

[B35] Costantini L. Magno S. Albanese D. Donati C. Molinari R. Filippone A.. (2018). Characterization of human breast tissue microbiota from core needle biopsies through the analysis of multi hypervariable 16S-rRNA gene regions. Sci. Rep. 8, 16893. doi: 10.1038/s41598-018-35329-z 30442969 PMC6237987

[B36] Costarelli V. Sanders T. A. B. (2002). Plasma deoxycholic acid concentration is elevated in postmenopausal women with newly diagnosed breast cancer. Eur. J. Clin. Nutr. 56, 925–927. doi: 10.1038/sj.ejcn.1601396 12209383

[B37] Creekmore B. C. Gray J. H. Walton W. G. Biernat K. A. Little M. S. Xu Y.. (2019). Mouse gut microbiome-encoded β-glucuronidases identified using metagenome analysis guided by protein structure. mSystems 4. doi: 10.1128/mSystems.00452-19 PMC6712278 31455640

[B38] Dalby M. J. Ross A.W. Walker A.W. Morgan P. J. (2017). Dietary uncoupling of gut microbiota and energy harvesting from obesity and glucose tolerance in mice. Cell Rep. 21, 1521–1533. doi: 10.1016/j.celrep.2017.10.056 29117558 PMC5695904

[B39] Dapito D. H. Mencin A. Gwak G. -Y. Pradere J. -P. Jang M. -K. Mederacke I.. (2012). Promotion of hepatocellular carcinoma by the intestinal microbiota and TLR4. Cancer Cell 21, 504–516. doi: 10.1016/j.ccr.2012.02.007 22516259 PMC3332000

[B40] d’Hennezel E. Abubucker S. Murphy L. O. Cullen T. W. (2017). Total lipopolysaccharide from the human gut microbiome silences toll-like receptor signaling. mSystems 2. doi: 10.1128/mSystems.00046-17 PMC5686520 29152585

[B41] Di Ciaula A. Wang D. Q. Molina-Molina E. Lunardi Baccetto R. Calamita G. Palmieri V. O.. (2017). Bile acids and cancer: direct and environmental-dependent effects. Ann. Hepatol. 16, s87–s105. doi: 10.5604/01.3001.0010.5501 29080344

[B42] Dwivedi A. K. Dubey P. Cistola D. P. Reddy S. Y. (2020). Association between obesity and cardiovascular outcomes: updated evidence from meta-analysis studies. Curr. Cardiol. Rep. 22, 25. doi: 10.1007/s11886-020-1273-y 32166448 PMC12285736

[B43] Errington J. (2013). L-form bacteria, cell walls and the origins of life. Open Biol. 3, 120143. doi: 10.1098/rsob.120143 23303308 PMC3603455

[B44] Ervin S. M. Li H. Lim L. Roberts L. R. Liang X. Mani S.. (2019). Gut microbial β-glucuronidases reactivate estrogens as components of the estrobolome that reactivate estrogens. J. Biol. Chem. 294, 18586–18599. doi: 10.1074/jbc.RA119.010950 31636122 PMC6901331

[B45] Esposito M. V. Fosso B. Nunziato M. Casaburi G. D'Argenio V. Calabrese A.. (2022). Microbiome composition indicate dysbiosis and lower richness in tumor breast tissues compared to healthy adjacent paired tissue, within the same women. BMC Cancer 22, 30. doi: 10.1186/s12885-021-09074-y 34980006 PMC8722097

[B46] Ewertz M. Jensen M. B. Gunnarsdottir K. A. Hojris I. Jakobsen E. H. Nielsen D.. (2011). Effect of obesity on prognosis after early-stage breast cancer. J. Clin. Oncol. 29, 25–31. doi: 10.1200/JCO.2010.29.7614 21115856

[B47] Feng Z. Hu Y. Wang X. Li Y. Yu Y. He J.. (2022). *In situ* imaging for tumor microbiome interactions via imaging mass cytometry on single-cell level. Cytometry A 101, 617–629. doi: 10.1002/cyto.a.24550 35301803

[B48] Fernández-Rojas B. Vázquez-Cervantes G. I. Pedraza-Chaverri J. Gutiérrez-Venegas G. (2020). Lipoteichoic acid reduces antioxidant enzymes in H9c2 cells. Toxicol. Rep. 7, 101–108. doi: 10.1016/j.toxrep.2019.12.007 31921600 PMC6948251

[B49] Flores R. Shi J. Fuhrman B. Xu X. Veenstra T. D. Gail M. H.. (2012). Fecal microbial determinants of fecal and systemic estrogens and estrogen metabolites: a cross-sectional study. J. Transl. Med. 10, 253. doi: 10.1186/1479-5876-10-253 23259758 PMC3552825

[B50] Fruge A. D. Van der Pol W. Rogers L. Q. Morrow C. D. Tsuruta Y. Demark-Wahnefried W. (2020). Fecal akkermansia muciniphila is associated with body composition and microbiota diversity in overweight and obese women with breast cancer participating in a presurgical weight loss trial. J. Acad. Nutr. Diet 120, 650–659. doi: 10.1016/j.jand.2018.08.164 30420171 PMC6509025

[B51] Fu A. Yao B. Dong T. Chen Y. Yao J. Liu Y.. (2022). Tumor-resident intracellular microbiota promotes metastatic colonization in breast cancer. Cell 185, 1356–1372.e26. doi: 10.1016/j.cell.2022.02.027 35395179

[B52] Fuhrman B. J. Feigelson H. S. Flores R. Gail M. H. Xu X. Ravel J.. (2014). Associations of the fecal microbiome with urinary estrogens and estrogen metabolites in postmenopausal women. J. Clin. Endocrinol. Metab. 99, 4632–4640. doi: 10.1210/jc.2014-2222 25211668 PMC4255131

[B53] Giaginis C. Karandrea D. Alexandrou P. Giannopoulou I. Tsourouflis G. Troungos C.. (2017). High Farnesoid X Receptor (FXR) expression is a strong and independent prognosticator in invasive breast carcinoma. Neoplasma 64, 633–639. doi: 10.4149/neo_2017_420 28485172

[B54] Goedert J. J. Hua X. Bielecka A. Okayasu I. Milne G. L. Jones G. S.. (2015). Investigation of the association between the fecal microbiota and breast cancer in postmenopausal women: a population-based case-control pilot study. J. Natl. Cancer Inst 107. doi: 10.1093/jnci/djv147 PMC4554191 26032724

[B55] Goedert J. J. Jones G. Hua X. Xu X. Yu G. Flores R.. (2018). Postmenopausal breast cancer and estrogen associations with the IgA-coated and IgA-noncoated faecal microbiota. Br. J. Cancer 118, 471–479. doi: 10.1038/bjc.2017.435 29360814 PMC5830593

[B56] Goldin B. R. Adlercreutz H. Gorbach S. L. Warram J. H. Dwyer J. T. Swenson L.. (1982). Estrogen excretion patterns and plasma levels in vegetarian and omnivorous women. New Engl. J. Med. 307, 1542–1547. doi: 10.1056/NEJM198212163072502 7144835

[B57] Gonzalez C. Williamson S. Gammon S. T. Glazer S. Rhee J. H. Piwnica-Worms D. (2023). TLR5 agonists enhance anti-tumor immunity and overcome resistance to immune checkpoint therapy. Commun. Biol. 6, 31. doi: 10.1038/s42003-022-04403-8 36635337 PMC9837180

[B58] González-Reyes S. Marín L. González L. González L. O. del Casar J. M. Lamelas M. L.. (2010). Study of TLR3, TLR4 and TLR9 in breast carcinomas and their association with metastasis. BMC Cancer 10, 665. doi: 10.1186/1471-2407-10-665 21129170 PMC3009680

[B59] Grivennikov S. I. Wang K. Mucida D. Stewart C. A. Schnabl B. Jauch D.. (2012). Adenoma-linked barrier defects and microbial products drive IL-23/IL-17-mediated tumor growth. Nature 491, 254–258. doi: 10.1038/nature11465 23034650 PMC3601659

[B60] Gunter M. J. Hoover D. R. Yu H. Wassertheil-Smoller S. Rohan T. E. Manson J. E.. (2009). Insulin, insulin-like growth factor-I, and risk of breast cancer in postmenopausal women. J. Natl. Cancer Inst 101, 48–60. doi: 10.1093/jnci/djn415 19116382 PMC2639294

[B61] Gunter M. J. Xie X. Xue X. Kabat G. C. Rohan T. E. Wassertheil-Smoller S.. (2015). Breast cancer risk in metabolically healthy but overweight postmenopausal women. Cancer Res. 75, 270–274. doi: 10.1158/0008-5472.CAN-14-2317 25593034 PMC4657855

[B62] Haeusler R. A. Astiarraga B. Camastra S. Accili D. Ferrannini E. (2013). Human insulin resistance is associated with increased plasma levels of 12α-hydroxylated bile acids. Diabetes 62, 4184–4191. doi: 10.2337/db13-0639 23884887 PMC3837033

[B63] Hansen N. W. Sams A. (2018). The microbiotic highway to health-new perspective on food structure, gut microbiota, and host inflammation. Nutrients 10. doi: 10.3390/nu10111590 PMC6267475 30380701

[B64] Hartung M. L. Gruber D. C. Koch K. N. Grüter L. Rehrauer H. Tegtmeyer N.. (2015). H. pylori-induced DNA strand breaks are introduced by nucleotide excision repair endonucleases and promote NF-κB target gene expression. Cell Rep. 13, 70–79. doi: 10.1016/j.celrep.2015.08.074 26411687

[B65] He C. Liu Y. Ye S. Yin S. Gu J. (2021). Changes of intestinal microflora of breast cancer in premenopausal women. Eur. J. Clin. Microbiol. Infect. Dis. 40, 503–513. doi: 10.1007/s10096-020-04036-x 32936397

[B66] Heikkinen S. Pitkäniemi J. Sarkeala T. Malila N. Koskenvuo M. (2015). Does hair dye use increase the risk of breast cancer? A population-based case-control study of finnish women. PloS One 10, e0135190. doi: 10.1371/journal.pone.0135190 26263013 PMC4532449

[B67] Heintz-Buschart A. Wilmes P. (2018). Human gut microbiome: function matters. Trends Microbiol. 26, 563–574. doi: 10.1016/j.tim.2017.11.002 29173869

[B68] Hieken T. J. Chen J. Hoskin T. L. Walther-Antonio M. Johnson S. Ramaker S.. (2016). The microbiome of aseptically collected human breast tissue in benign and Malignant disease. Sci. Rep. 6, 30751. doi: 10.1038/srep30751 27485780 PMC4971513

[B69] Himbert C. Delphan M. Scherer D. Bowers L. W. Hursting S. Ulrich C. M. (2017). Signals from the adipose microenvironment and the obesity-cancer link-A systematic review. Cancer Prev. Res. (Phila) 10, 494–506. doi: 10.1158/1940-6207.CAPR-16-0322 28864539 PMC5898450

[B70] Hossain F. Majumder S. David J. Bunnell B. A. Miele L. (2021). Obesity modulates the gut microbiome in triple-negative breast cancer. Nutrients 13. doi: 10.3390/nu13103656 PMC8539565 34684657

[B71] Hou M. F. Ou-Yang F. Li C. L. Chen F. M. Chuang C. H. Kan J. Y.. (2021). Comprehensive profiles and diagnostic value of menopausal-specific gut microbiota in premenopausal breast cancer. Exp. Mol. Med. 53, 1636–1646. doi: 10.1038/s12276-021-00686-9 34707191 PMC8569190

[B72] Hsieh H. L. Lin C. C. Shih R. H. Hsiao L. D. Yang C. M. (2012). NADPH oxidase-mediated redox signal contributes to lipoteichoic acid-induced MMP-9 upregulation in brain astrocytes. J. Neuroinflamm. 9, 110. doi: 10.1186/1742-2094-9-110 PMC3391180 22643046

[B73] I.A.R.C. (2023). Agents classified by the IARC monographs Vol. 1–135 (Lyon: IARC). Available at: http://monographs.iarc.fr/ENG/Classification/index.php.

[B74] Inan M. S. Rasoulpour R. J. Yin L. Hubbard A.K. Rosenberg D. W. Giardina C. (2000). The luminal short-chain fatty acid butyrate modulates NF-kappaB activity in a human colonic epithelial cell line. Gastroenterology 118, 724–734. doi: 10.1016/S0016-5085(00)70142-9 10734024

[B75] Iwase T. Sangai T. Nagashima T. Sakakibara M. Sakakibara J. Hayama S.. (2016). Impact of body fat distribution on neoadjuvant chemotherapy outcomes in advanced breast cancer patients. Cancer Med. 5, 41–48. doi: 10.1002/cam4.571 26626021 PMC4708907

[B76] Jaeggi T. Kortman G. A. Moretti D. Chassard C. Holding P. Dostal A.. (2015). Iron fortification adversely affects the gut microbiome, increases pathogen abundance and induces intestinal inflammation in Kenyan infants. Gut 64, 731–742. doi: 10.1136/gutjnl-2014-307720 25143342

[B77] Javitt N. B. Budai K. Raju U. Levitz M. Miller D. G. Cahan A.C. (1994). Breast-gut connection: origin of chenodeoxycholic acid in breast cyst fluid. Lancet 343, 633–635. doi: 10.1016/S0140-6736(94)92635-2 7906811

[B78] Jia Y. Hong J. Li H. Hu Y. Jia L. Cai D.. (2017). Butyrate stimulates adipose lipolysis and mitochondrial oxidative phosphorylation through histone hyperacetylation-associated β3-adrenergic receptor activation in high-fat diet-induced obese mice. Exp. Physiol. 102, 273–281. doi: 10.1113/EP086114 28028849

[B79] Jiang Y. Gong W. Xian Z. Xu W. Hu J. Ma Z.. (2023). 16S full-length gene sequencing analysis of intestinal flora in breast cancer patients in Hainan Province. Mol. Cell Probes 71, 101927. doi: 10.1016/j.mcp.2023.101927 37595804

[B80] Kers J. G. Saccenti E. (2021). The power of microbiome studies: some considerations on which alpha and beta metrics to use and how to report results. Front. Microbiol. 12, 796025. doi: 10.3389/fmicb.2021.796025 35310396 PMC8928147

[B81] Kikuchi K. Saigusa D. Kanemitsu Y. Matsumoto Y. Thanai P. Suzuki N.. (2019). Gut microbiome-derived phenyl sulfate contributes to albuminuria in diabetic kidney disease. Nat. Commun. 10, 1835. doi: 10.1038/s41467-019-09735-4 31015435 PMC6478834

[B82] Kim H. J. Kim S. R. Park J. K. Kim D. I. Jeong J. S. Lee Y. C. (2012). PI3Kγ activation is required for LPS-induced reactive oxygen species generation in respiratory epithelial cells. Inflammation Res. 61, 1265–1272. doi: 10.1007/s00011-012-0526-7 22825625

[B83] Kirschner S. K. Ghane P. Park J. K. Simbo S. Y. Ivanov I. Braga-Neto U. M.. (2023). Short-chain fatty acid production in accessible and inaccessible body pools as assessed by novel stable tracer pulse approach is reduced by aging independent of presence of COPD. Metabolism 141, 155399. doi: 10.1016/j.metabol.2023.155399 36642114

[B84] Klann E. Williamson J. M. Tagliamonte M. S. Ukhanova M. Asirvatham J. R. Chim H.. (2020). Microbiota composition in bilateral healthy breast tissue and breast tumors. Cancer Causes Control 31, 1027–1038. doi: 10.1007/s10552-020-01338-5 32844256

[B85] Kovacs T. Miko E. Vida A. Sebo E. Toth J. Csonka T.. (2019). Cadaverine, a metabolite of the microbiome, reduces breast cancer aggressiveness through trace amino acid receptors. Sci. Rep. 9, 1300. doi: 10.1038/s41598-018-37664-7 30718646 PMC6361949

[B86] Krishnamurthy K. Wang G. Rokhfeld D. Bieberich E. (2008). Deoxycholate promotes survival of breast cancer cells by reducing the level of pro-apoptotic ceramide. Breast Cancer Res. 10, R106. doi: 10.1186/bcr2211 19087284 PMC2656903

[B87] Krych L. Hansen C. H. Hansen A.K. van den Berg F. W. Nielsen D. S. (2013). Quantitatively different, yet qualitatively alike: a meta-analysis of the mouse core gut microbiome with a view towards the human gut microbiome. PloS One 8, e62578. doi: 10.1371/journal.pone.0062578 23658749 PMC3641060

[B88] Lauby-Secretan B. Scoccianti C. Loomis D. Grosse Y. Bianchini F. Straif K. (2016). Body fatness and cancer–viewpoint of the IARC working group. N Engl. J. Med. 375, 794–798. doi: 10.1056/NEJMsr1606602 27557308 PMC6754861

[B89] Le Chatelier E. Nielsen T. Qin J. Prifti E. Hildebrand F. Falony G.. (2013). Richness of human gut microbiome correlates with metabolic markers. Nature 500, 541–546. doi: 10.1038/nature12506 23985870

[B90] Lemaitre R. N. Jensen P. N. Wang Z. Fretts A.M. McKnight B. Nemet I.. (2021). Association of trimethylamine N-oxide and related metabolites in plasma and incident type 2 diabetes: the cardiovascular health study. JAMA Netw. Open 4, e2122844. doi: 10.1001/jamanetworkopen.2021.22844 34448864 PMC8397925

[B91] Le May N. Mota-Fernandes D. Vélez-Cruz R. Iltis I. Biard D. Egly J. M. (2010). NER factors are recruited to active promoters and facilitate chromatin modification for transcription in the absence of exogenous genotoxic attack. Mol. Cell 38, 54–66. doi: 10.1016/j.molcel.2010.03.004 20385089

[B92] Le May N. Fradin D. Iltis I. Bougnères P. Egly J. M. (2012). XPG and XPF endonucleases trigger chromatin looping and DNA demethylation for accurate expression of activated genes. Mol. Cell 47, 622–632. doi: 10.1016/j.molcel.2012.05.050 22771116

[B93] Ley R. E. Bäckhed F. Turnbaugh P. Lozupone C. A. Knight R. D. Gordon J. I. (2005). Obesity alters gut microbial ecology. Proc. Natl. Acad. Sci. 102, 11070–11075. doi: 10.1073/pnas.0504978102 16033867 PMC1176910

[B94] Ley R. E. Turnbaugh P. J. Klein S. Gordon J. I. (2006). Human gut microbes associated with obesity. Nature 444, 1022–1023. doi: 10.1038/4441022a 17183309

[B95] Li T. Chiang J. Y. (2014). Bile acid signaling in metabolic disease and drug therapy. Pharmacol. Rev. 66, 948–983. doi: 10.1124/pr.113.008201 25073467 PMC4180336

[B96] Li S. Xu X. Jiang M. Bi Y. Xu J. Han M. (2015). Lipopolysaccharide induces inflammation and facilitates lung metastasis in a breast cancer model via the prostaglandin E2-EP2 pathway. Mol. Med. Rep. 11, 4454–4462. doi: 10.3892/mmr.2015.3258 25625500

[B97] Liang G. Bushman F. D. (2021). The human virome: assembly, composition and host interactions. Nat. Rev. Microbiol. 19, 514–527. doi: 10.1038/s41579-021-00536-5 33785903 PMC8008777

[B98] Liu C. H. Chen Z. Chen K. Liao F. T. Chung C. E. Liu X.. (2021). Lipopolysaccharide-mediated chronic inflammation promotes tobacco carcinogen-induced lung cancer and determines the efficacy of immunotherapy. Cancer Res. 81, 144–157. doi: 10.1158/0008-5472.CAN-20-1994 33122306 PMC7878420

[B99] Liu Y. Hu Y. Xue J. Li J. Yi J. Bu J.. (2023). Advances in immunotherapy for triple-negative breast cancer. Mol. Cancer 22, 145. 37660039 10.1186/s12943-023-01850-7 PMC10474743

[B100] Loftfield E. Herzig K. H. Caporaso J. G. Derkach A. Wan Y. Byrd D. A.. (2020). Association of body mass index with fecal microbial diversity and metabolites in the northern Finland birth cohort. Cancer Epidemiol. Biomarkers Prev. 29, 2289–2299. doi: 10.1158/1055-9965.EPI-20-0824 32855266 PMC7642019

[B101] Lohmann A. E. Goodwin P. J. Chlebowski R. T. Pan K. Stambolic V. Dowling R. J. (2016). Association of obesity-related metabolic disruptions with cancer risk and outcome. J. Clin. Oncol. 34, 4249–4255. doi: 10.1200/JCO.2016.69.6187 27903146

[B102] Loman B. R. Russart K. L.G. Grant C. V. Lynch A.J. Bailey M. T. Pyter L. M. (2022). Mammary tumors alter the fecal bacteriome and permit enteric bacterial translocation. BMC Cancer 22, 245. doi: 10.1186/s12885-022-09274-0 35248004 PMC8897840

[B103] Long S. L. Gahan C. G. M. Joyce S. A. (2017). Interactions between gut bacteria and bile in health and disease. Mol. Aspects Med. 56, 54–65. doi: 10.1016/j.mam.2017.06.002 28602676

[B104] Lumachi F. Santeufemia D. A. Basso S. M. (2015). Current medical treatment of estrogen receptor-positive breast cancer. World J. Biol. Chem. 6, 231–239. doi: 10.4331/wjbc.v6.i3.231 26322178 PMC4549764

[B105] Luu T. H. Michel C. Bard J. M. Dravet F. Nazih H. Bobin-Dubigeon C. (2017). Intestinal proportion of blautia sp. is *associated with clinical stage and histoprognostic grade in patients with early-stage breast cancer* . Nutr. Cancer 69, 267–275. doi: 10.1080/01635581.2017.1263750 28094541

[B106] Ma J. Sun L. Liu Y. Ren H. Shen Y. Bi F.. (2020). Alter between gut bacteria and blood metabolites and the anti-tumor effects of Faecalibacterium prausnitzii in breast cancer. BMC Microbiol. 20, 82. doi: 10.1186/s12866-020-01739-1 32272885 PMC7144064

[B107] Ma Z. Qu M. Wang X. (2022). Analysis of gut microbiota in patients with breast cancer and benign breast lesions. Pol. J. Microbiol. 71, 217–226. doi: 10.33073/pjm-2022-019 35675827 PMC9252143

[B108] Martinez-Medina M. Aldeguer X. Gonzalez-Huix F. Acero D. Garcia-Gil L. J. (2006). Abnormal microbiota composition in the ileocolonic mucosa of Crohn’s disease patients as revealed by polymerase chain reaction-denaturing gradient gel electrophoresis. Inflammation Bowel Dis. 12, 1136–1145. doi: 10.1097/01.mib.0000235828.09305.0c 17119388

[B109] Martinot E. Sèdes L. Baptissart M. Lobaccaro J. M. Caira F. Beaudoin C.. (2017). Bile acids and their receptors. Mol. Aspects Med. 56, 2–9. doi: 10.1016/j.mam.2017.01.006 28153453

[B110] Marzullo P. Bettini S. Menafra D. Aprano S. Muscogiuri G. Barrea L.. (2021). Spot-light on microbiota in obesity and cancer. Int. J. Obes. (Lond) 45, 2291–2299. doi: 10.1038/s41366-021-00866-7 34363002

[B111] Mehmeti M. Allaoui R. Bergenfelz C. Saal L. H. Ethier S. P. Johansson M. E.. (2015). Expression of functional toll like receptor 4 in estrogen receptor/progesterone receptor-negative breast cancer. Breast Cancer Res. 17, 130. doi: 10.1186/s13058-015-0640-x 26392082 PMC4578669

[B112] Miko E. Vida A. Kovacs T. Ujlaki G. Trencsenyi G. Marton J.. (2018). Lithocholic acid, a bacterial metabolite reduces breast cancer cell proliferation and aggressiveness. Biochim. Biophys. Acta Bioenerg 1859, 958–974. doi: 10.1016/j.bbabio.2018.04.002 29655782

[B113] Minelli E. B. Beghini A.M. Vesentini S. Marchiori L. Nardo G. Cerutti R.. (1990). Intestinal microflora as an alternative metabolic source of estrogens in women with uterine leiomyoma and breast cancer. Ann. New York Acad. Sci. 595, 473–479. doi: 10.1111/j.1749-6632.1990.tb34337.x

[B114] Mishra R. Rajsiglová L. Lukáč P. Tenti P. Šima P. Čaja F.. (2021). Spontaneous and induced tumors in germ-free animals: A general review. Medicina (Kaunas) 57. doi: 10.3390/medicina57030260 PMC8002107 33799911

[B115] Molinaro A. Bel Lassen P. Henricsson M. Wu H. Adriouch S. Belda E.. (2020). Author Correction: Imidazole propionate is increased in diabetes and associated with dietary patterns and altered microbial ecology. Nat. Commun. 11, 6448. doi: 10.1038/s41467-020-20412-9 33349634 PMC7752903

[B116] Muller M. Hernandez M. A.G. Goossens G. H. Reijnders D. Holst J. J. Jocken J. W.E.. (2019). Circulating but not fecal short-chain fatty acids are related to insulin sensitivity, lipolysis and GLP-1 concentrations in humans. Sci. Rep. 9, 12515. 31467327 10.1038/s41598-019-48775-0 PMC6715624

[B117] Murphy K. Weaver C. Janeway C. (2017). Janeway's immunobiology (New York: Garland Science).

[B118] Nagpal R. Shively C. A. Appt S. A. Register T. C. Michalson K. T. Vitolins M. Z.. (2018). Gut microbiome composition in non-human primates consuming a western or mediterranean diet. Front. Nutr. 5, 28. doi: 10.3389/fnut.2018.00028 29922651 PMC5996930

[B119] Nearing J. T. Comeau A. M. Langille M. G. I. (2021). Identifying biases and their potential solutions in human microbiome studies. Microbiome 9, 113. doi: 10.1186/s40168-021-01059-0 34006335 PMC8132403

[B120] Nejman D. Livyatan I. Fuks G. Gavert N. Zwang Y. Geller L. T.. (2020). The human tumor microbiome is composed of tumor type-specific intracellular bacteria. Science 368, 973–980. doi: 10.1126/science.aay9189 32467386 PMC7757858

[B121] Newman T. M. Shively C. A. Register T. C. Appt S. E. Yadav H. Colwell R. R.. (2021). Diet, obesity, and the gut microbiome as determinants modulating metabolic outcomes in a non-human primate model. Microbiome 9, 100. doi: 10.1186/s40168-021-01069-y 33952353 PMC8101030

[B122] Núñez Abad M. Calabuig-Fariñas S. Lobo de Mena M. Torres-Martínez S. García González C. García García J.. (2022). Programmed death-ligand 1 (PD-L1) as immunotherapy biomarker in breast cancer. Cancers (Basel) 14. doi: 10.3390/cancers14020307 PMC8773553 35053471

[B123] Ollberding N. J. Kim Y. Shvetsov Y. B. Wilkens L. R. Franke A.A. Cooney R. V.. (2013). Prediagnostic leptin, adiponectin, C-reactive protein, and the risk of postmenopausal breast cancer. Cancer Prev. Res. (Phila) 6, 188–195. doi: 10.1158/1940-6207.CAPR-12-0374 23466816 PMC3595121

[B124] Pan K. Chlebowski R. T. Mortimer J. E. Gunther M. J. Rohan T. Vitolins M. Z.. (2020). Insulin resistance and breast cancer incidence and mortality in postmenopausal women in the Women’s Health Initiative. Cancer 126, 3638–3647. doi: 10.1002/cncr.33002 32530506

[B125] Panigrahi G. Candia J. Dorsey T. H. Tang W. Ohara Y. Byun J. S.. (2023). Diabetes-associated breast cancer is molecularly distinct and shows a DNA damage repair deficiency. JCI Insight. doi: 10.1172/jci.insight.170105 PMC10795835 37906280

[B126] Parada Venegas D. De la Fuente M. K. Landskron G. Gonzalez M. J. Quera R. Dijkstra G.. (2019). Short chain fatty acids (SCFAs)-mediated gut epithelial and immune regulation and its relevance for inflammatory bowel diseases. Front. Immunol. 10, 277. doi: 10.3389/fimmu.2019.00277 30915065 PMC6421268

[B127] Parida S. Siddharth S. Gatla H. R. Wu S. Wang G. Gabrielson K.. (2021). A procarcinogenic colon microbe promotes breast tumorigenesis and metastatic progression and concomitantly activates notch and β-catenin axes. Cancer Discovery 11, 1138–1157. doi: 10.1158/2159-8290.CD-20-0537 33408241

[B128] Parida S. Wu S. Siddharth S. Wang G. Muniraj N. Nagalingam A.. (2023). Gut colonization with an obesity-associated enteropathogenic microbe modulates the premetastatic niches to promote breast cancer lung and liver metastasis. Front. Immunol. 14, 1194931. doi: 10.3389/fimmu.2023.1194931 37503343 PMC10369066

[B129] Parida S. Sharma D. (2019). The microbiome-estrogen connection and breast cancer risk. Cells 8. doi: 10.3390/cells8121642 PMC6952974 31847455

[B130] Park G. S. Kim J. H. (2015). Myeloid differentiation primary response gene 88-leukotriene B4 receptor 2 cascade mediates lipopolysaccharide-potentiated invasiveness of breast cancer cells. Oncotarget 6, 5749–5759. doi: 10.18632/oncotarget.v6i8 25691060 PMC4467399

[B131] Pearce K. L. Hill A. Tremellen K. P. (2019). Obesity related metabolic endotoxemia is associated with oxidative stress and impaired sperm DNA integrity. Basic Clin. Androl 29, 6. doi: 10.1186/s12610-019-0087-5 31114691 PMC6513521

[B132] Pendyala S. Walker J. M. Holt P. R. (2012). A high-fat diet is associated with endotoxemia that originates from the gut. Gastroenterology 142, 1100–1101.e2. doi: 10.1053/j.gastro.2012.01.034 22326433 PMC3978718

[B133] Peters B. A. Lin J. Qi Q. Usyk M. Isasi C. R. Mossavar-Rahmani Y.. (2022). Menopause is associated with an altered gut microbiome and estrobolome, with implications for adverse cardiometabolic risk in the hispanic community health study/study of Latinos. mSystems 7, e0027322. doi: 10.1128/msystems.00273-22 35675542 PMC9239235

[B134] Peters B. A. Kelly L. Wang T. Loudig O. Rohan T. E.. (2023). The breast microbiome in breast cancer risk and progression: a narrative review. Cancer Epidemiol. Biomarkers Prev. 10.1158/1055-9965.EPI-23-0965 37943168

[B135] Picon-Ruiz M. Morata-Tarifa C. Valle-Goffin J. J. Friedman E. R. Slingerland J. M. (2017). Obesity and adverse breast cancer risk and outcome: Mechanistic insights and strategies for intervention. CA Cancer J. Clin. 67, 378–397. doi: 10.3322/caac.21405 28763097 PMC5591063

[B136] Plottel C. S. Blaser M. J. (2011). Microbiome and Malignancy. Cell Host Microbe 10, 324–335. doi: 10.1016/j.chom.2011.10.003 22018233 PMC3264051

[B137] Poggi M. Bastelica D. Gual P. Iglesias M. A. Gremeaux T. Knauf C.. (2007). C3H/HeJ mice carrying a toll-like receptor 4 mutation are protected against the development of insulin resistance in white adipose tissue in response to a high-fat diet. Diabetologia 50, 1267–1276. doi: 10.1007/s00125-007-0654-8 17426960

[B138] Raftogianis R. Creveling C. Weinshilboum R. Weisz J. (2000). Chapter 6: estrogen metabolism by conjugation. JNCI Monogr. 2000, 113–124. doi: 10.1093/oxfordjournals.jncimonographs.a024234 10963623

[B139] Ridlon J. M. Kang D. J. Hylemon P. B. (2006). Bile salt biotransformations by human intestinal bacteria. J. Lipid Res. 47, 241–259. doi: 10.1194/jlr.R500013-JLR200 16299351

[B140] Rigiracciolo D. C. Scarpelli A. Lappano R. Pisano A. Santolla M. F. Avino S.. (2016). GPER is involved in the stimulatory effects of aldosterone in breast cancer cells and breast tumor-derived endothelial cells. Oncotarget 7, 94–111. doi: 10.18632/oncotarget.v7i1 26646587 PMC4807985

[B141] Rock C. L. Flatt S. W. Byers T. E. Colditz G. A. Demark-Wahnefried W. Ganz P. A.. (2015). Results of the exercise and nutrition to enhance recovery and good health for you (ENERGY) trial: A behavioral weight loss intervention in overweight or obese breast cancer survivors. J. Clin. Oncol. 33, 3169–3176. doi: 10.1200/JCO.2015.61.1095 26282657 PMC4582146

[B142] Rowland I. Gibson G. Heinken A. Scott K. Swann J. Thiele I.. (2018). Gut microbiota functions: metabolism of nutrients and other food components. Eur. J. Nutr. 57, 1–24. doi: 10.1007/s00394-017-1445-8 PMC5847071 28393285

[B143] Roy K. Kozłowski H. M. Jędrzejewski T. Sobocińska J. Maciejewski B. Dzialuk A.. (2023). Endotoxin tolerance creates favorable conditions for cancer development. Cancers (Basel) 15. doi: 10.3390/cancers15205113 PMC10605812 37894480

[B144] Rutkowski M. R. Stephen T. L. Svoronos N. Allegrezza M. J. Tesone A.J. Perales-Puchalt A.. (2015). Microbially driven TLR5-dependent signaling governs distal Malignant progression through tumor-promoting inflammation. Cancer Cell 27, 27–40. doi: 10.1016/j.ccell.2014.11.009 25533336 PMC4293269

[B145] Saad M. J. Santos A. Prada P. O. (2016). Linking gut microbiota and inflammation to obesity and insulin resistance. Physiol. (Bethesda) 31, 283–293. doi: 10.1152/physiol.00041.2015 27252163

[B146] Sacksteder M. R. (1976). Occurrence of spontaneous tumors in the germfree F344 rat. J. Natl. Cancer Inst 57, 1371–1373. doi: 10.1093/jnci/57.6.1371 1069860

[B147] Santos-Marcos J. A. Rangel-Zuniga O. A. Jimenez-Lucena R. Quintana-Navarro G. M. Garcia-Carpintero S. Malagon M. M.. (2018). Influence of gender and menopausal status on gut microbiota. Maturitas 116, 43–53. doi: 10.1016/j.maturitas.2018.07.008 30244778

[B148] Sári Z. Mikó E. Kovács T. Boratkó A. Ujlaki G. Jankó L.. (2020a). Indolepropionic acid, a metabolite of the microbiome, has cytostatic properties in breast cancer by activating AHR and PXR receptors and inducing oxidative stress. Cancers (Basel) 12. doi: 10.3390/cancers12092411 PMC7565149 32854297

[B149] Sári Z. Mikó E. Kovács T. Jankó L. Csonka T. Lente G.. (2020b). Indoxylsulfate, a metabolite of the microbiome, has cytostatic effects in breast cancer via activation of AHR and PXR receptors and induction of oxidative stress. Cancers (Basel) 12. doi: 10.3390/cancers12102915 PMC7599465 33050543

[B150] Scheeren F. A. Kuo A.H. van Weele L. J. Cai S. Glykofridis I. Sikandar S. S.. (2014). A cell-intrinsic role for TLR2-MYD88 in intestinal and breast epithelia and oncogenesis. Nat. Cell Biol. 16, 1238–1248. doi: 10.1038/ncb3058 25362351

[B151] Scheithauer T. P. M. Rampanelli E. Nieuwdorp M. Vallance B. A. Verchere C. B. van Raalte D. H.. (2020). Gut microbiota as a trigger for metabolic inflammation in obesity and type 2 diabetes. Front. Immunol. 11. doi: 10.3389/fimmu.2020.571731 PMC7596417 33178196

[B152] Schreiber H. Nettesheim P. Lijinsky W. Richter C. B. Walburg H. E. (1972). Induction of lung cancer in germfree, specific-pathogen-free, and infected rats by N-nitrosoheptamethyleneimine: enhancement by respiratory infection. J. Natl. Cancer Inst 49, 1107–1114. 5084122

[B153] Schwabe R. F. Jobin C. (2013). The microbiome and cancer. Nat. Rev. Cancer 13, 800–812. doi: 10.1038/nrc3610 24132111 PMC3986062

[B154] Sears C. L. Geis A. L. Housseau F. (2014). Bacteroides fragilis subverts mucosal biology: from symbiont to colon carcinogenesis. J. Clin. Invest. 124, 4166–4172. doi: 10.1172/JCI72334 25105360 PMC4191034

[B155] Seol M. A. Park J. H. Jeong J. H. Lyu J. Han S. Y. Oh S. M. (2017). Role of TOPK in lipopolysaccharide-induced breast cancer cell migration and invasion. Oncotarget 8, 40190–40203. doi: 10.18632/oncotarget.v8i25 28212583 PMC5522254

[B156] Shakiba Y. Vorobyev P. O. Naumenko V. A. Kochetkov D. V. Zajtseva K. V. Valikhov M. P.. (2023). Oncolytic efficacy of a recombinant vaccinia virus strain expressing bacterial flagellin in solid tumor models. Viruses 15. doi: 10.3390/v15040828 PMC10142208 37112810

[B157] Shen W. D. Lin X. Liu H. M. Li B. Y. Qiu X. Lv W. Q.. (2022). Gut microbiota accelerates obesity in peri-/post-menopausal women via Bacteroides fragilis and acetic acid. Int. J. Obes. (Lond) 46, 1918–1924. doi: 10.1038/s41366-022-01137-9 35978102

[B158] Shi M. Yao Y. Han F. Li Y. Li Y. (2014). MAP1S controls breast cancer cell TLR5 signaling pathway and promotes TLR5 signaling-based tumor suppression. PloS One 9, e86839. doi: 10.1371/journal.pone.0086839 24466264 PMC3900661

[B159] Shinozaki S. Choi C. S. Shimizu N. Yamada M. Kim M. Zhang T.. (2011). Liver-specific inducible nitric-oxide synthase expression is sufficient to cause hepatic insulin resistance and mild hyperglycemia in mice. J. Biol. Chem. 286, 34959–34975. doi: 10.1074/jbc.M110.187666 21846719 PMC3186386

[B160] Shively C. A. Register T. C. Appt S. E. Clarkson T. B. Uberseder B. Clear K. Y.J.. (2018). Consumption of mediterranean versus western diet leads to distinct mammary gland microbiome populations. Cell Rep. 25, 47–56.e3. doi: 10.1016/j.celrep.2018.08.078 30282037 PMC6338220

[B161] Shrode R. L. Knobbe J. E. Cady N. Yadav M. Hoang J. Cherwin C.. (2023). Breast cancer patients from the Midwest region of the United States have reduced levels of short-chain fatty acid-producing gut bacteria. Sci. Rep. 13, 526. doi: 10.1038/s41598-023-27436-3 36631533 PMC9834383

[B162] Shuang C. Weiguang Y. Zhenkun F. Yike H. Jiankun Y. Jing X.. (2017). Toll-like receptor 5 gene polymorphism is associated with breast cancer susceptibility. Oncotarget 8, 88622–88629. doi: 10.18632/oncotarget.v8i51 29179462 PMC5687632

[B163] Smith A. Pierre J. F. Makowski L. Tolley E. Lyn-Cook B. Lu L.. (2019). Distinct microbial communities that differ by race, stage, or breast-tumor subtype in breast tissues of non-Hispanic Black and non-Hispanic White women. Sci. Rep. 9, 11940. doi: 10.1038/s41598-019-48348-1 31420578 PMC6697683

[B164] Smith K. S. Fruge A.D. van der Pol W. Caston N. E. Morrow C. D. Demark-Wahnefried W.. (2021). Gut microbial differences in breast and prostate cancer cases from two randomized controlled trials compared to matched cancer-free controls. Benef Microbes 12, 239–248. doi: 10.3920/BM2020.0098 33789551 PMC8328038

[B165] Smith A. Gu Q. Amos-Abanyie E. K. Tolley E. Lu L. Lyn-Cook B.. (2022). Abstract 3022: Tryptophan metabolism is associated with obesity and triple negative breast cancer risk in black and white women. Cancer Res. 82, 3022–3022. doi: 10.1158/1538-7445.AM2022-3022

[B166] Sokol H. Pigneur B. Watterlot L. Lakhdari O. Bermúdez-Humarán L. G. Gratadoux J. J.. (2008). Faecalibacterium prausnitzii is an anti-inflammatory commensal bacterium identified by gut microbiota analysis of Crohn disease patients. Proc. Natl. Acad. Sci. U.S.A. 105, 16731–16736. doi: 10.1073/pnas.0804812105 18936492 PMC2575488

[B167] Soto-Pantoja D. R. Gaber M. Arnone A. A. Bronson S. M. Cruz-Diaz N. Wilson A.S.. (2021). Diet alters entero-mammary signaling to regulate the breast microbiome and tumorigenesis. Cancer Res. 81, 3890. doi: 10.1158/0008-5472.CAN-20-2983 34083249 PMC8981494

[B168] Tan J. McKenzie C. Potamitis M. Thorburn A.N. Mackay C. R. Macia L. (2014). The role of short-chain fatty acids in health and disease. Adv. Immunol. 121, 91–119. doi: 10.1016/B978-0-12-800100-4.00003-9 24388214

[B169] Tang W. Putluri V. Ambati C. R. Dorsey T. H. Putluri N. Ambs S. (2019). Liver- and microbiome-derived bile acids accumulate in human breast tumors and inhibit growth and improve patient survival. Clin. Cancer Res. 25, 5972–5983. doi: 10.1158/1078-0432.CCR-19-0094 31296531 PMC6774910

[B170] Tenvooren I. Jenks M. Z. Rashid H. Cook K. L. Muhlemann J. K. Sistrunk C.. (2019). Elevated leptin disrupts epithelial polarity and promotes premalignant alterations in the mammary gland. Oncogene 38, 3855–3870. doi: 10.1038/s41388-019-0687-8 30670780 PMC6525037

[B171] Thangaraju M. Cresci G. A. Liu K. Ananth S. Gnanaprakasam J. P. Browning D. D.. (2009). GPR109A is a G-protein-coupled receptor for the bacterial fermentation product butyrate and functions as a tumor suppressor in colon. Cancer Res. 69, 2826–2832. doi: 10.1158/0008-5472.CAN-08-4466 19276343 PMC3747834

[B172] Thompson K. J. Ingle J. N. Tang X. Chia N. Jeraldo P. R. Walther-Antonio M. R.. (2017). A comprehensive analysis of breast cancer microbiota and host gene expression. PloS One 12, e0188873. doi: 10.1371/journal.pone.0188873 29190829 PMC5708741

[B173] Tilg H. Kaser A. (2011). Gut microbiome, obesity, and metabolic dysfunction. J. Clin. Invest. 121, 2126–2132. doi: 10.1172/JCI58109 21633181 PMC3104783

[B174] Toumazi D. El Daccache S. Constantinou C. (2021). An unexpected link: The role of mammary and gut microbiota on breast cancer development and management (Review). Oncol. Rep. 45, 80. doi: 10.3892/or 33786630

[B175] Turnbaugh P. J. Ley R. E. Mahowald M. A. Magrini V. Mardis E. R. Gordon J. I. (2006). An obesity-associated gut microbiome with increased capacity for energy harvest. Nature 444, 1027–1031. doi: 10.1038/nature05414 17183312

[B176] Turnbaugh P. J. Backhed F. Fulton L. Gordon J. I. (2008). Diet-induced obesity is linked to marked but reversible alterations in the mouse distal gut microbiome. Cell Host Microbe 3, 213–223. doi: 10.1016/j.chom.2008.02.015 18407065 PMC3687783

[B177] Tzeng A. Sangwan N. Jia M. Liu C. C. Keslar K. S. Downs-Kelly E.. (2021). Human breast microbiome correlates with prognostic features and immunological signatures in breast cancer. Genome Med. 13, 60. doi: 10.1186/s13073-021-00874-2 33863341 PMC8052771

[B178] Urbaniak C. Cummins J. Brackstone M. Macklaim J. M. Gloor G. B. Baban C. K.. (2014). Microbiota of human breast tissue. Appl. Environ. Microbiol. 80, 3007–3014. doi: 10.1128/AEM.00242-14 24610844 PMC4018903

[B179] Urbaniak C. Gloor G. B. Brackstone M. Scott L. Tangney M. Reid G. (2016). The microbiota of breast tissue and its association with breast cancer. Appl. Environ. Microbiol. 82, 5039–5048. doi: 10.1128/AEM.01235-16 27342554 PMC4968547

[B180] Vatanen T. Kostic A.D. d'Hennezel E. Siljander H. Franzosa E. A. Yassour M.. (2016). Variation in microbiome LPS immunogenicity contributes to autoimmunity in humans. Cell 165, 842–853. doi: 10.1016/j.cell.2016.04.007 27133167 PMC4950857

[B181] Vidi P. -A. Bissell M. J. Lelièvre S. A. (2013). Three-dimensional culture of human breast epithelial cells: the how and the why. Methods Mol. Biol. (Clifton N.J.) 945, 193–219. 10.1007/978-1-62703-125-7_13 PMC3666567 23097109

[B182] Wei M. Huang F. Zhao L. Zhang Y. Yang W. Wang S.. (2020). A dysregulated bile acid-gut microbiota axis contributes to obesity susceptibility. eBioMedicine 55. doi: 10.1016/j.ebiom.2020.102766 PMC7225614 32408110

[B183] W.H.O (2021) Obesity and overweight. Available online at: https://www.who.int/news-room/fact-sheets/detail/obesity-and-overweight.

[B184] Włodarczyk M. Nowicka G. (2019). Obesity, DNA damage, and development of obesity-related diseases. Int. J. Mol. Sci. 20. doi: 10.3390/ijms20051146 PMC6429223 30845725

[B185] Wu A. H. Tseng C. Vigen C. Yu Y. Cozen W. Garcia A.A.. (2020). Gut microbiome associations with breast cancer risk factors and tumor characteristics: a pilot study. Breast Cancer Res. Treat 182, 451–463. doi: 10.1007/s10549-020-05702-6 32468338 PMC7297869

[B186] Xiong R. G. Zhou D. D. Wu S. X. Huang S. Y. Saimaiti A. Yang Z. J.. (2022). Health benefits and side effects of short-chain fatty acids. Foods 11. doi: 10.3390/foods11182863 PMC9498509 36140990

[B187] Xu Z. Jiang W. Huang W. Lin Y. Chan F. K.L. Ng S. C. (2022). Gut microbiota in patients with obesity and metabolic disorders — a systematic review. Genes Nutr. 17, 2. doi: 10.1186/s12263-021-00703-6 35093025 PMC8903526

[B188] Xuan C. Shamonki J. M. Chung A. Dinome M. L. Chung M. Sieling P. A.. (2014). Microbial dysbiosis is associated with human breast cancer. PloS One 9, e83744–e83744. doi: 10.1371/journal.pone.0083744 24421902 PMC3885448

[B189] Yang H. Wang B. Wang T. Xu L. He C. Wen H.. (2014). Toll-like receptor 4 prompts human breast cancer cells invasiveness via lipopolysaccharide stimulation and is overexpressed in patients with lymph node metastasis. PloS One 9, e109980. doi: 10.1371/journal.pone.0109980 25299052 PMC4192367

[B190] Yang P. Wang Z. Peng Q. Lian W. Chen D. (2021). Comparison of the gut microbiota in patients with benign and Malignant breast tumors: A pilot study. Evol. Bioinform. Online 17, 11769343211057573. doi: 10.1177/11769343211057573 34795472 PMC8593289

[B191] Yassine F. Fostok S. F. Al Deen N. N. Talhouk R. S. (2021). Endotoxin triggers tumor initiation events in nontumorigenic breast epithelial cells and enhances invasion-related phenotype in pretumorigenic and tumorigenic breast epithelial cells. Int. J. Inflam 2021, 4666380. doi: 10.1155/2021/4666380 PMC8642002 34868543

[B192] Yoshimoto S. Loo T. M. Atarashi K. Kanda H. Sato S. Oyadomari S.. (2013). Obesity-induced gut microbial metabolite promotes liver cancer through senescence secretome. Nature 499, 97–101. doi: 10.1038/nature12347 23803760

[B193] Zhang F. Aschenbrenner D. Yoo J. Y. Zuo T. (2022). The gut mycobiome in health, disease, and clinical applications in association with the gut bacterial microbiome assembly. Lancet Microbe 3, e969–e983. doi: 10.1016/S2666-5247(22)00203-8 36182668

[B194] Zhou H. Hylemon P. B. (2014). Bile acids are nutrient signaling hormones. Steroids 86, 62–68. doi: 10.1016/j.steroids.2014.04.016 24819989 PMC4073476

[B195] Zhu J. Liao M. Yao Z. Liang W. Li Q. Liu J.. (2018). Breast cancer in postmenopausal women is associated with an altered gut metagenome. Microbiome 6, 136. doi: 10.1186/s40168-018-0515-3 30081953 PMC6080540

